# Iron Metabolism Disorders Associated With the Severity of Pyogenic Liver Abscess

**DOI:** 10.1002/fsn3.71195

**Published:** 2025-11-21

**Authors:** Zhongyu Han, Zhenchao Wu, Han Zhou, Taikang Yao, Fan Jiang, Yingqiu Ying, Ming Lu, Zihe Zhou, Zilu Wang, Ning Shen, Jiajia Zheng

**Affiliations:** ^1^ Department of Laboratory Medicine Peking University Third Hospital Beijing China; ^2^ Department of Pulmonary and Critical Care Medicine Peking University Third Hospital Beijing China; ^3^ Department of Pharmacy Peking University Third Hospital Beijing China; ^4^ Center for Infectious Diseases Peking University Third Hospital Beijing China

**Keywords:** bacterial infection, dietary nutrition, ferritin, iron metabolism, liver abscess

## Abstract

Pyogenic liver abscess (PLA) is a serious intra‐abdominal infection with high morbidity and mortality. Current diagnostic methods are time‐consuming, inefficient and high‐cost. Rapid identification/assessment of PLA and early recognition of bacterial liver abscess (BLA) improve prognosis. Iron metabolism disorders play a critical role in infections, but their role in PLA (especially ferritin) remains unclear. Notably, dietary nutrition, as a modulator of iron metabolism/immune function, may influence PLA progression, with limited research. In this study, we collected clinical data of 125 PLA patients from the Medical Information Mart for Intensive Care IV (MIMIC‐IV) database to identify rapid, low‐cost severity biomarkers, and verified them by an independent clinical cohort of 36 patients from Peking University Third Hospital. We analyzed the correlation between iron metabolism and disease severity, explored independent risk factors, and evaluated diagnostic efficiency via receiver operating characteristic (ROC) curves. Iron metabolism disorders were observed in patients with PLA and correlated strongly with liver dysfunction and systemic inflammation. Ferritin was associated with disease severity and served as an independent prognostic marker, with optimal cut‐offs of 390 ng/mL for predicting severe PLA and 748 ng/mL for identifying BLA. This study identifies ferritin as a promising multifaceted biomarker for PLA which not only reflects disease severity but also aids in the early identification of bacterial etiology. Additionally, the findings provide a basis for dietary nutritional interventions in PLA management.

## Graphical abstract

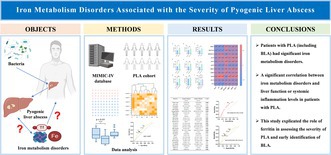

**Abbreviations**
AG: anion gap
ALB: albumin
ALBI: albumin–bilirubin score
ALP: alkaline phosphatase
ALT: alanine aminotransferase
AMY: amylase
APRI: aspartate aminotransferase to platelet ratio index
APTT: activated partial thromboplastin time
AST: aspartate transaminase
BC: bicarbonate
BLA: bacterial liver abscess
BUN: blood urea nitrogen
Ca: calcium
CI: confidence interval
CK: creatine kinase
CK‐MB: creatine kinase muscle and brain isoenzyme
Cl: chloride
CNPLA: culture negative pyogenic liver abscess
CR: creatinine
DBIL: direct bilirubin
FIB: fibrinogen
GLB: globulin
GLU: glucose
HCT: hematocrit
HGB: hemoglobin
I‐Bil: indirect bilirubin
INR: international normalized ratio
K: potassium
LDH: lactate dehydrogenase
MELD: model for end—stage liver disease
MONO#: monocyte count
MONO%: monocyte ratio
Na: sodium
NEUT#: neutrophil count
NEUT%: neutrophil ratio
OR: odds ratio
PLA: pyogenic liver abscess
PLT: platelet
PT: prothrombin time
RBC: red blood cell
TBIL: total bilirubin
TF: transferrin
TIBC: total iron binding capacity
TP: total protein
WBC: white blood cell

## 1 Introduction

Liver abscess is a digestive system infection caused by pathogens (e.g., bacteria, fungi, amoebae) that invade the liver via the biliary tract, hepatic artery, or portal vein, leading to focal purulent lesions in the liver. In severe cases, it can result in hepatocyte necrosis, liver function impairment, and high levels of inflammation, and even high mortality (Khim et al. 2019; López‐Cano Gómez et al. 2012). Pyogenic liver abscess (PLA) is the most common type, accounting for approximately 80% of all liver abscesses, with a mortality rate as high as about 30% (Shelat et al. 2015). Moreover, the incidence of PLA is increasing year by year (Chen et al. 2016). PLA is characterized by its acute onset and nonspecific clinical manifestations, commonly presenting with high fever, chills, and right upper quadrant pain (Serraino et al. 2018). These symptoms can easily be confused with other diseases, thus increasing the difficulty of diagnosis. Therefore, exploring biomarkers for assessing the severity of PLA can help identify severe PLA and facilitate early diagnosis and treatment. Once pathogens disseminate from the liver into the bloodstream, they can easily cause multi‐organ infection, leading to prolonged treatment duration, increased antibiotic usage, significantly increased economic burden and poor prognosis (Siu et al. 2012). Currently, the etiological diagnosis of PLA primarily relies on the culture of aspirated fluid, which is invasive, time‐consuming, and associated with low diagnostic yield. The development of serum biomarkers capable of early identifying bacterial infections is essential to facilitate prompt and targeted treatment, thereby preventing the occurrence of invasion and improving therapeutic outcome.

Iron, as a trace element, plays an important role in metabolism. The continuous process of iron absorption, utilization, storage and circulation makes iron dynamic balance, which runs through the whole iron homeostasis. The liver acts as the most predominant organ in charge of iron homeostasis, modulating the synthesis of ferritin to maintain this balance (Anderson and Frazer 2017). A study found patients with nonalcoholic fatty liver disease (NAFLD) had higher serum ferritin levels, which were positively correlated with the degree of hepatic steatosis (Zelber‐Sagi et al. 2007). In addition, ferritin can also be used as a marker of liver injury caused by COVID‐19 (Da et al. 2021). In infectious diseases, iron is an essential element for pathogens and hosts to maintain life. The competition for iron resources between pathogens and hosts is central to survival during the infection process (Holt et al. 2015). More and more studies prove serum ferritin is closely related to the development of infectious disease. For instance, serum ferritin has been shown to predict prognosis in dengue and influenza infections (Lalueza et al. 2020; Shukla et al. 2023), while disturbances in iron metabolism have been linked to heightened susceptibility to bacterial/viral pneumonia and sepsis (Wu et al. 2025). Although PLA is a severe intra‐abdominal infection with high morbidity and mortality, our comprehension of the etiology and pathophysiology of PLA actually remains rather rudimentary. The role of iron metabolism in disease development, especially for ferritin, remains largely unknown. Notably, the homeostasis of iron metabolism in the body, which is related to dietary nutrition and nutritional immune mechanisms, may affect the absorption and utilization of iron and the intensity of inflammatory responses. However, there is currently no research on its potential role in the development of PLA.

Therefore, this study aims to investigate the correlation between iron metabolism disorders and PLA severity, explore the effectiveness of iron biomarkers in assessing PLA severity and identifying bacterial infections, and provide a theoretical basis for explaining the role of nutritional immune mechanisms in the occurrence and development of PLA.

## 2 Materials and Methods

### 2.1 Source of Data

The clinical data in this retrospective study were extracted from the Medical Information Mart for Intensive Care IV (MIMIC‐IV 1.0 version) database which recorded comprehensive clinical information from patients admitted to the emergency department or ICU at Beth Israel Deaconess Medical Center in Boston from 2008 to 2019 (Johnson et al. 2023). Access to the data was facilitated by completing the National Institutes of Health (NIH) curriculum on human subject study protection and successfully passing the Collaborative Institutional Training Initiative (CITI) program assessments.

### 2.2 Study Population

The study focused on patients with pyogenic liver abscess. We selected 493 patients with pyogenic liver abscess by standard codes of the 9th version of the International Classification of Diseases (ICD‐9): 572.0 and the 10th version of the International Classification of Diseases (ICD‐10): K75.0. Patients were excluded if they met any of the following criteria: (1) bacterial culture and identification were not conducted (*n* = 78); (2) the results of serum iron, transferrin, total iron binding capacity, and ferritin were all missing (*n* = 290). Ultimately, a total of 125 patients from MIMIC‐IV were included in our analysis.

This study enrolled a clinical cohort consisting of 36 patients with PLA and 10 healthy controls from Peking University Third Hospital, and systematically collected their laboratory test results and serum samples for targeted analyses.

### 2.3 Observational Variables

Data extraction was performed using structured query language (SQL). Based on the study purpose and clinical practice, comprehensive data regarding each patient were collected, such as: (1) demographic information, including gender and age; (2) laboratory indicators, including iron metabolism indicators: serum iron, transferrin (TF), total iron binding capacity (TIBC) and ferritin; erythroid indicators: red blood cell (RBC), hemoglobin (HGB) and hematocrit (HCT); inflammatory indicators: white blood cell (WBC), neutrophil ratio (NEUT%) and monocyte ratio (MONO%); liver function indicators: alanine aminotransferase (ALT), aspartate transaminase (AST), alkaline phosphatase (ALP), total bilirubin (TBIL), lactate dehydrogenase (LDH) and albumin (ALB); coagulation indicators: platelet (PLT), international normalized ratio (INR), prothrombin time (PT) and activated partial thromboplastin time (APTT); other biochemical indicators: blood urea nitrogen (BUN), creatinine (CR) and glucose (GLU); pathogen identification results. Liver function scores: Model for End–Stage Liver Disease (MELD), albumin–bilirubin Score (ALBI), and aspartate aminotransferase to platelet ratio index (APRI) are calculated through the above‐mentioned indicators (Kamath and Kim 2007; Toyoda and Johnson 2022; Yilmaz et al. 2011). The reference intervals of observation indicators were shown in Table S1. Taking the test time of the lowest serum iron as the reference point, match the results of other examination items that are closest within 48 h. Items with missing values greater than 20% are excluded. Items with missing values less than or equal to 20% are imputed using the mean value. Through sensitivity analysis, it was found that the dataset after imputation is stable, reliable and representative (Tables S2 and S3). Therefore, in order to avoid result bias, the original unimputed data is used for analysis.

The mild PLA was defined as patients with alanine aminotransferase (ALT) and white blood cell (WBC) both normal, moderate PLA was defined as cases with elevation of either ALT or WBC, and severe PLA was defined as patients with ALT and WBC both rising (Du et al. 2016; Kwo et al. 2017; Zhu et al. 2011). Furthermore, according to the pathogen identification results, the patients were divided into bacterial liver abscess (BLA) and culture‐negative pyogenic liver abscess (CNPLA) to perform subgroup analysis (Shelat et al. 2016).

### 2.4 Detection of Serum Iron Metabolism Indicators

The concentrations of serum iron and TIBC were determined by AU5800 Series Clinical Chemistry Analyzers (Beckman Coulter, USA) according to the manufacturer's instructions. The concentrations of ferritin were determined by cobas e801 analytical unit (Roche Diagnostics, USA) according to the manufacturer's instructions.

### 2.5 Statistical Analysis

Statistical analysis was performed on SPSS 26.0 (IBM, USA) and GraphPad Prism 9 (GraphPad Software, USA) software. The correlation analysis was conducted by Spearman correlation. Continuous variables' normality was tested by Shapiro—Wilk. Normal ones were presented as mean ± SD and compared with Independent Samples *t*—test or one‐way ANOVA; non‐ normal ones as median (IQR) and compared by Mann–Whitney *U* test. For continuous variables with multiple independent non‐normal distributions, the Kruskal–Wallis *H* test is used. Categorical variables were expressed as frequencies (*n*) and percentages (%), and the chi‐square test was used for comparison between groups.

Before regression analysis, variables with *p* < 0.05 were first identified via univariate screening. Then, considering statistical power and clinical relevance, indicators strongly correlated with the grouping variable (WBC/NEUT%/ALT/AST/ALP/TBIL) were excluded, and the remaining variables were included in the multivariate model. The Receiver Operating Characteristic (ROC) curve was plotted. The diagnostic efficacy of ferritin was evaluated by the Area Under the Curve (AUC). The optimal cut‐off value was found based on the maximum value of the Youden index. A *p*‐value < 0.05 indicated a significant difference.

## 3 Results

### 3.1 Investigate the Clinical Characteristics and Microbiological Features in Patients With PLA

**FIGURE 1:**
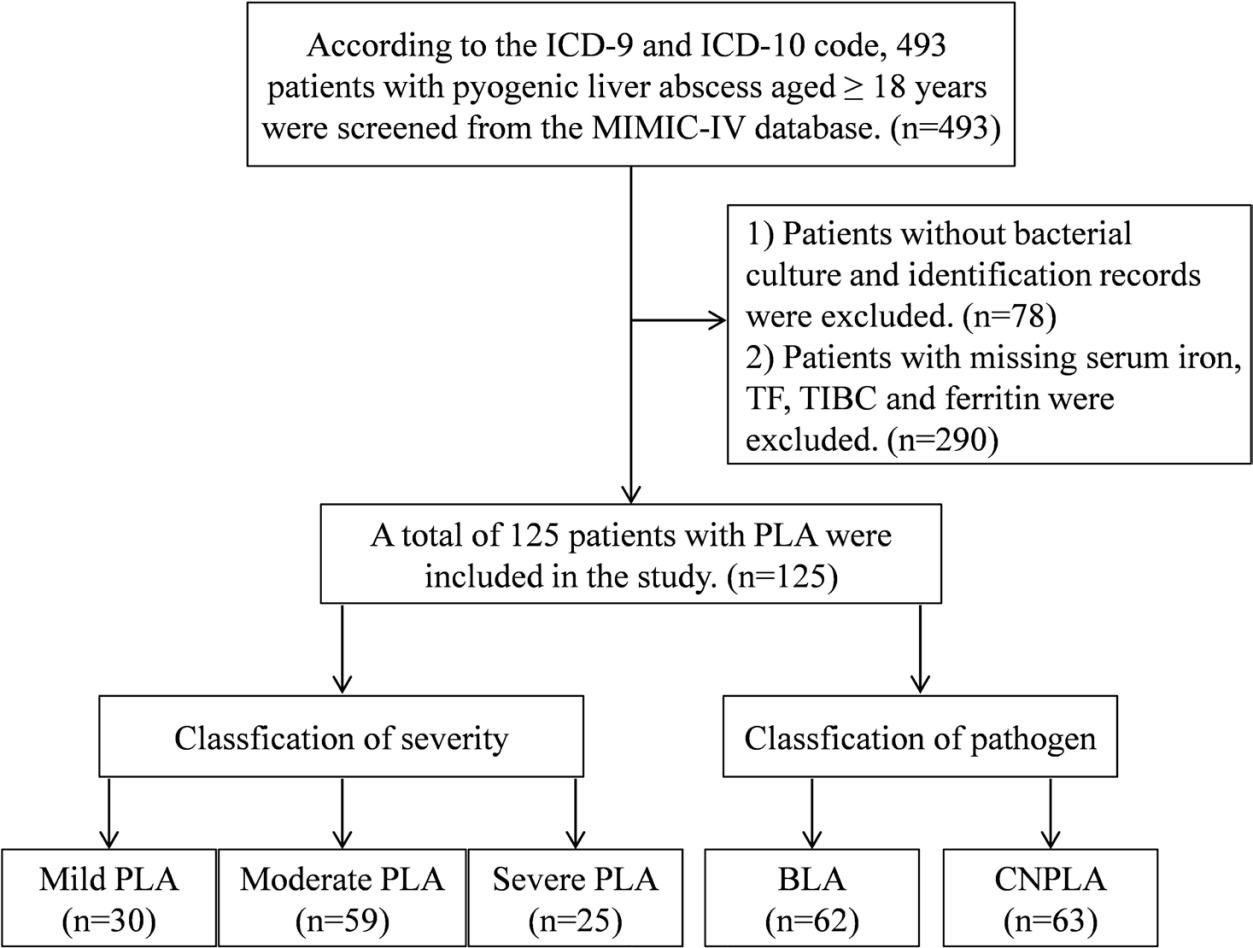
Flowchart of patient selection process for pyogenic liver abscess (PLA) from the MIMIC‐IV database. Patients with pyogenic liver abscess were selected by standard codes of the 9th version of the international classification of diseases (ICD‐9): 572.0 and the 10th version of the international classification of diseases (ICD‐10): K75.0. Patients were excluded if they met any of the following criteria: (1) age < 18 years; (2) bacterial culture and identification were not conducted; (3) the results of serum iron, transferrin, total iron binding capacity, and ferritin were all missing. Data extraction was performed using structured query language (SQL). Data imputation was conducted using adjacent values within 24/48 h.

In this retrospective study, a total of 125 patients with PLA who met the inclusion criteria were screened from 493 patients with pyogenic liver abscess (Figure 1). The ages of patients spanned from 22 to 91 years old, which included 59.2% males (*n* = 74) and 40.8% females (*n* = 51) (Table 1). The patients with PLA had increased WBC and decreased RBC and HGB. Notably, they had significant iron metabolism disorders, such as, decreased serum iron, TF and TIBC, and increased ferritin. Based on pathogen identification results, 62 patients were diagnosed with bacterial liver abscess (BLA), including 37.10% (*n* = 23) infection with Gram‐positive bacteria, 61.29% (*n* = 38) with Gram‐negative bacterial infection, and 1.61% (*n* = 1) with mixed bacterial flora (Figure S1a). *Escherichia coli* (13, 20.97%) and *Klebsiella pneumoniae* (9, 14.52%) were the dominant pathogens (Figure S1b).

**TABLE 1:** Baseline characteristics of all patients.

Indicators	All patients (*n* = 125)	Reference intervals
Gender, male	74.0 (59.2%)	—
Age, years	62.0 ± 13.5	—
Iron (μg/dL)	22.0 (13.0, 33.5) ↓	45.0–160.0 (male) 30.0–160.0 (female)
TF (mg/dL)	146.3 ± 44.3 ↓	200.0–360.0
TIBC (μg/dL)	190.2 ± 57.5 ↓	260.0–470.0
Ferritin (ng/mL)	519.5 (264.0, 937.5) ↑	30.0–400.0 (male) 13.0–150.0 (female)
RBC (m/μL)	3.3 (2.9, 3.6)	4.6–6.2
HGB (g/dL)	9.2 (8.0, 10.4)	14.0–18.0
HCT (%)	28.6 (25.6, 31.8)	40.0–52.0
WBC (K/μL)	11.1 (7.4, 16.3)	4.0–11.0
NEUT% (%)	81.5 (73.0, 88.0)	50.0–70.0
MONO% (%)	5.3 (3.7, 7.3)	2.0–11.0
ALT (IU/L)	32.0 (17.8, 64.5)	0.0–40.0
AST (IU/L)	32.5 (20.8, 54.0)	0.0–40.0
ALP (IU/L)	161.5 (109.3, 278.0)	40.0–130.0
TBIL (mg/dL)	0.7 (0.4, 1.3)	0.0–1.5
LDH (IU/L)	246.5 (186.3, 254.8)	94.0–250.0
ALB (g/dL)	2.8 ± 0.5	3.5–5.2
PLT (K/μL)	288.0 (156.0, 419.5)	150.0–440.0
INR (−)	1.3 (1.2, 1.5)	0.9–1.1
PT (sec)	14.6 (13.3, 16.4)	9.4–12.5
APTT (sec)	30.3 (28.0, 33.9)	25.0–36.5
BUN (mg/dL)	14.5 (10.0, 22.3)	6.0–20.0
CR (mg/dL)	0.9 (0.6, 1.2)	0.4–1.1
GLU (mg/dL)	119.5 (96.0, 156.3)	70.0–100.0
MELD score	10.0 (7.0, 15.5)	—
ALBI score	−1.5 ± 0.6	—
APRI score	0.3 (0.2, 1.1)	—

Abbreviations: ALB, albumin; ALBI, albumin–bilirubin score; ALP, alkaline phosphatase; ALT, alanine aminotransferase; APRI, Aspartate Aminotransferase to Platelet Ratio Index; APTT, activated partial thromboplastin time; AST, aspartate transaminase; BUN, blood urea nitrogen; CR, creatinine; GLU, glucose; HCT, hematocrit; HGB, hemoglobin; INR, international normalized ratio; LDH, lactate dehydrogenase; MELD, model for end–stage liver disease; MONO%, monocyte ratio; NEUT%, neutrophil ratio; PLA, pyogenic liver abscess; PLT, platelet; PT, prothrombin time; RBC, red blood cell; TBIL, total bilirubin; TF, transferrin; TIBC, total iron binding capacity; WBC, white blood cell.

### 3.2 Explore the Correlation Between Iron Metabolism Disorders and Severity in Patients With PLA

**FIGURE 2:**
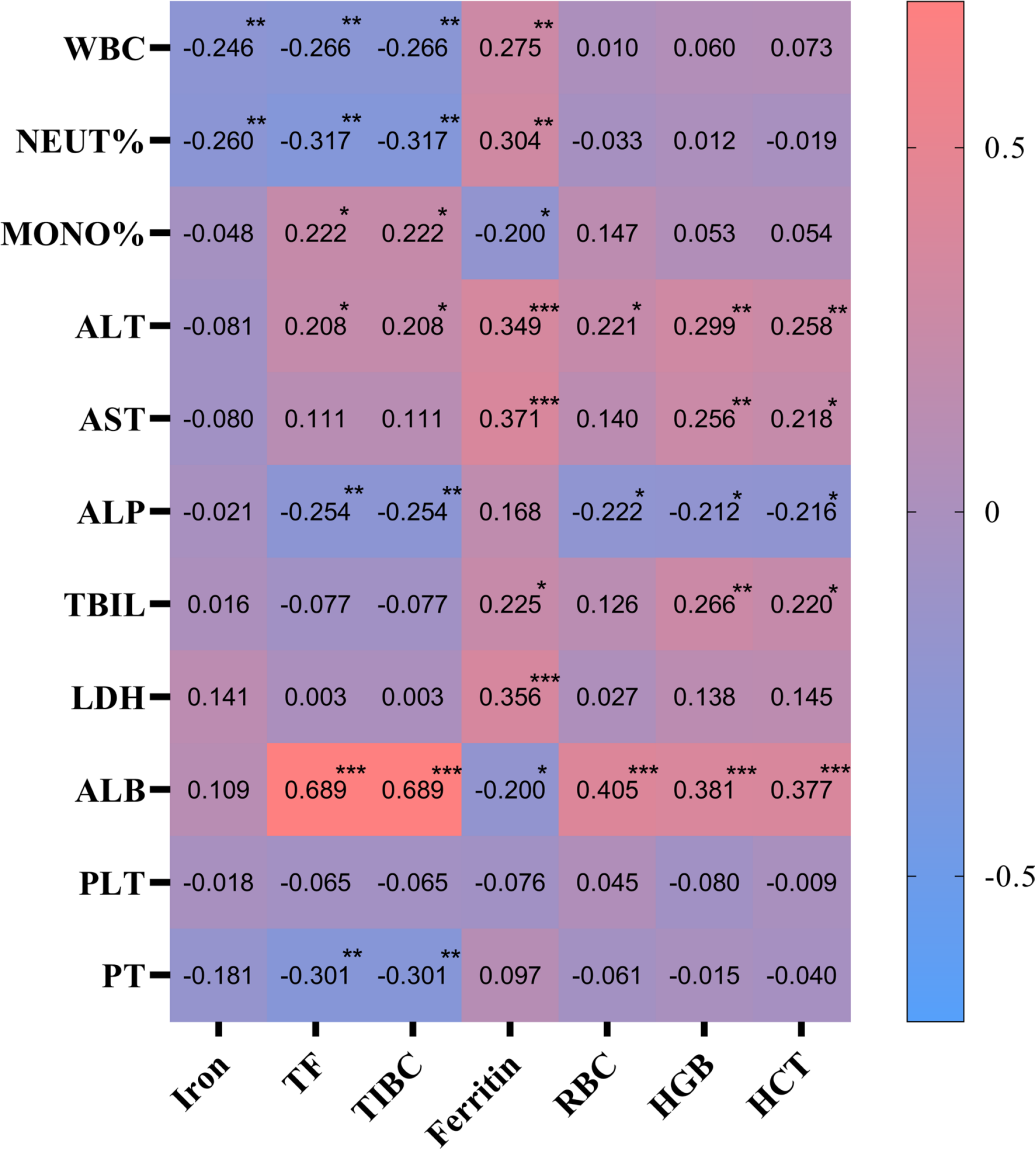
Correlation heat map of iron metabolism indicators with liver function and systemic inflammation level in patients with PLA. ALB, albumin; ALP, alkaline phosphatase; ALT, alanine aminotransferase; AST, aspartate transaminase; HCT, hematocrit; HGB, hemoglobin; LDH, lactate dehydrogenase; MONO%, monocyte ratio; NEUT%, neutrophil ratio; PLA, pyogenic liver abscess; PLT, platelet; PT, prothrombin time; RBC, red blood cell; TBIL, total bilirubin; TF, transferrin; TIBC, total iron binding capacity; WBC, white blood cell; *p < 0.05; **p < 0.01; ***p < 0.001.

**FIGURE 3:**
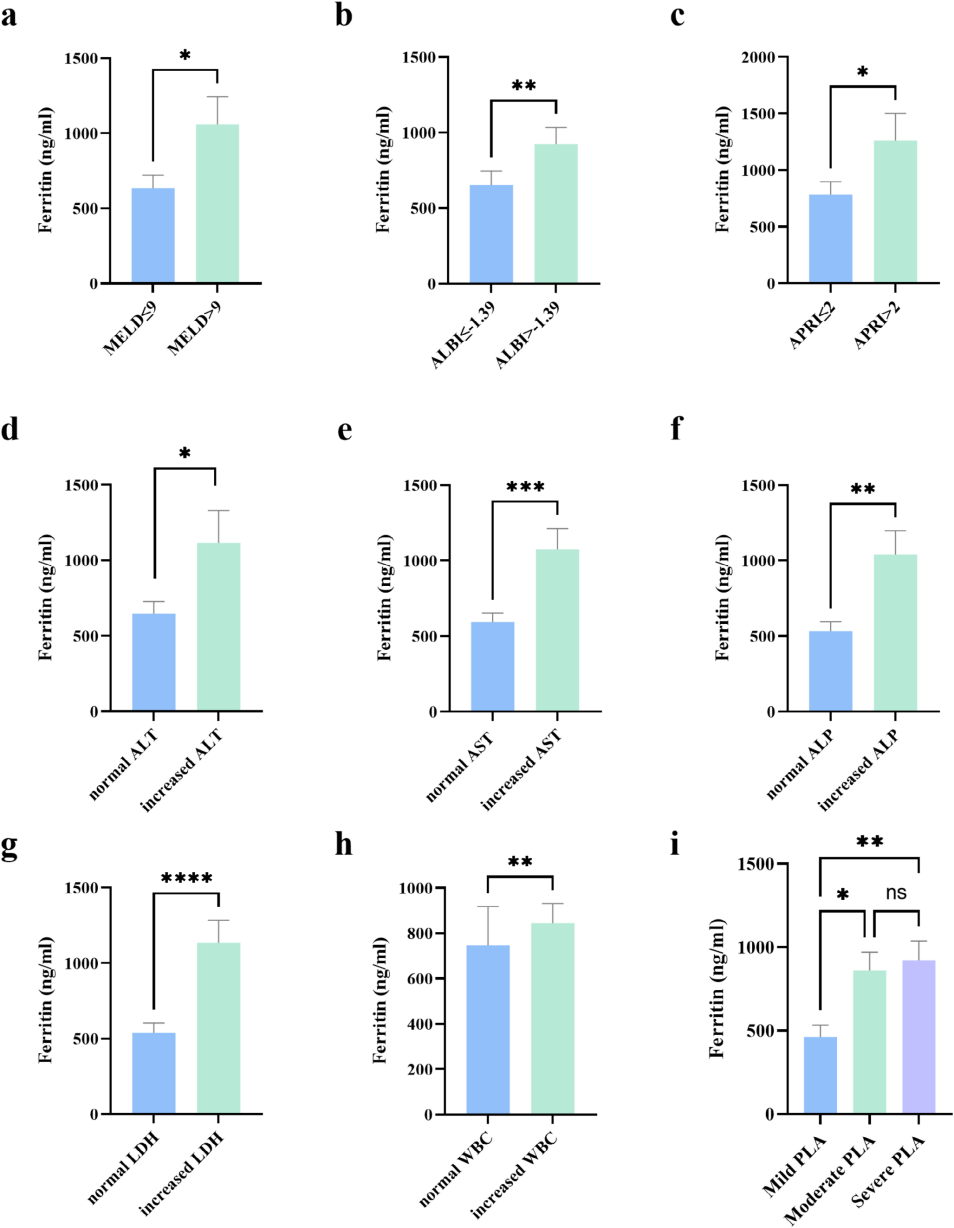
Serum ferritin levels across subgroups of patients with PLA. (a) Serum ferritin levels in patients with normal vs. increased MELD score; (b) serum ferritin levels in patients with normal vs. increased ALBI score; (c) serum ferritin levels in patients with normal vs. increased APRI score; (d) serum ferritin levels in patients with normal vs. increased ALT; (e) serum ferritin levels in patients with normal vs. increased AST; (f) serum ferritin levels in patients with normal vs. increased ALP; (g) serum ferritin levels in patients with normal vs. increased LDH; (h) serum ferritin levels in patients with normal vs. increased WBC; (f) serum ferritin levels in mild PLA, moderate PLA, and severe PLA. ALBI, albumin–bilirubin score; ALP, alkaline phosphatase; ALT, alanine aminotransferase; APRI, aspartate aminotransferase to platelet ratio index; AST, aspartate transaminase; LDH, lactate dehydrogenase; MELD, model for end—stage liver disease; PLA, pyogenic liver abscess; WBC, white blood cell. *p < 0.05; **p < 0.01; ***p < 0.001.

Serum iron was negatively correlated with WBC and NEUT%; TF and TIBC were positively correlated with ALT, ALB and MONO% and negatively correlated with ALP, PT, WBC and NEUT%; ferritin was positively correlated with ALT, AST, LDH, TBIL, WBC and NEUT% and negatively correlated with ALB and MONO% (Table S4). These results revealed a significant correlation between iron metabolism disorders in patients with PLA and liver function as well as systemic inflammation levels (Figure 2). Liver function and blood routine are crucial indicators for assessing the clinical status in patients with PLA. We conducted subgroup analysis from the perspective of liver function (liver function score: MELD/ALBI/APRI; liver parenchymal injury: ALT/AST/ALP/LDH; bilirubin metabolism: TBIL; hepatic synthesis function: ALB/PT) and systemic inflammation levels (WBC/NEUT%/MONO%) respectively (Tables S5–S17). Serum iron decreased in ALB, PT, WBC and NEUT% analysis; TF decreased in ALBI, ALP, ALB, WBC and NEUT% analysis; TIBC decreased in ALBI, ALP, ALB, WBC and NEUT% analysis; ferritin increased in MELD, ALBI, APRI, ALT, AST, ALP, LDH and WBC analysis (Figure 3a–h); HGB decreased in APRI and ALB analysis. Comprehensively, ferritin was generally related to liver function and systemic inflammation levels.

### 3.3 Explicit Ferritin as a Biomarker Reflecting on the Severity of PLA

ALT and WBC are widely acknowledged as reliable biomarkers in assessing liver function and systemic inflammation levels respectively (Emergency Medicine Branch of the Chinese Medical Association 2022; Du et al. 2016; Kwo et al. 2017; Senior 2012; Zhu et al. 2011). Based on the levels of ALT and WBC, the patients were stratified into three groups: mild, moderate, and severe. A comparative analysis of these three groups was conducted, revealing that among the three PLA groups, significant differences were observed in the indicators of iron, ferritin, WBC, NEUT%, ALT, AST, ALP, TBIL, ALB, ALBI score and APRI score. Specifically, as the disease severity increased, patients showed an upward trend in ferritin levels, systemic inflammation levels (WBC, NEUT%), and liver function markers (ALT, AST, ALP, TBIL), as well as the ALBI and APRI scores, while iron levels and ALB exhibited a decreasing trend (Table 2 and Figure 3i).

**TABLE 2:** Difference analysis of indicators among three PLA subgroups.

Indicators	Mild PLA (*n* = 30)	Moderate PLA (*n* = 59)	Severe PLA (*n* = 25)	*p*
Gender, male	16.0 (53.3%)	34.0 (57.6%)	18.0 (72.0%)	0.336
Age, years	62.8 ± 15.5	61.2 ± 12.1	62.0 ± 16.4	0.875
Iron (μg/dL)	30.0 (20.0, 43.0)	19.0 (12.5, 33.0)	17.0 (11.0, 24.0)	**0.009**
Iron_cat (decreased)	20.0 (66.6%)	42.0 (71.1%)	23.0 (92.0%)	**0.015**
TF (mg/dL)	154.9 ± 50.7	137.9 ± 43.6	139.4 ± 27.5	0.326
TIBC (μg/dL)	201.3 ± 66.0	179.2 ± 56.7	181.3 ± 35.8	0.330
Ferritin (ng/mL)	310.5 (169.8, 822.8)	560.0 (314.0, 1077.0)	832.5 (510.0, 1102.8)	**0.002**
Ferritin_cat (increased)	18.0 (60.0%)	46.0 (77.9%)	23.0 (92.0%)	**0.015**
RBC (m/μL)	3.2 (2.8, 3.5)	3.2 (2.8, 3.7)	3.4 (3.1, 3.8)	0.270
HGB (g/dL)	8.9 (7.7, 10.4)	9.0 (7.9, 10.3)	10.1 (8.9, 11.4)	0.061
HCT (%)	28.1 (25.0, 30.7)	28.0 (25.4, 31.3)	30.7 (27.5, 34.0)	0.114
WBC (K/μL)	7.3 ± 2.2	12.5 ± 5.7	18.2 ± 6.4	**< 0.001**
WBC_cat (increased)	0.0 (0%)	37.0 (62.7%)	25.0 (100%)	**< 0.001**
NEUT% (%)	75.0 (61.6, 83.1)	81.6 (73.9, 88.0)	89.0 (81.5, 90.7)	**< 0.001**
NEUT%_cat (increased)	16.0 (53.3%)	42.0 (71.1%)	21.0 (84.0%)	**0.014**
MONO% (%)	6.6 ± 3.7	5.4 ± 3.3	5.2 ± 3.8	0.288
ALT (IU/L)	19.0 (13.8, 25.3)	31.0 (17.0, 58.0)	69.0 (54.5, 93.5)	**< 0.001**
ALT_cat (increased)	0.0 (0%)	22.0 (37.2%)	25.0 (100%)	**< 0.001**
AST (IU/L)	22.0 (16.8, 29.0)	33.0 (21.0, 50.0)	70.0 (38.0, 123.5)	**< 0.001**
AST_cat (increased)	2.0 (6.6%)	24.0 (40.6%)	18.0 (72.0%)	**< 0.001**
ALP (IU/L)	103.0 (80.8, 149.3)	197.0 (136.0, 315.0)	198.0 (124.5, 368.0)	**< 0.001**
ALP_cat (increased)	9.0 (30.0%)	46.0 (77.9%)	18.0 (72.0%)	**< 0.001**
TBIL (mg/dL)	0.4 (0.3, 0.9)	0.7 (0.4, 1.5)	1.3 (0.7, 1.9)	**0.001**
TBIL_cat (increased)	3.0 (10.0%)	14.0 (23.7%)	8.0 (32.0%)	0.147
LDH (IU/L)	214.0 (156.5, 252.5)	220.5 (166.8, 266.8)	252.0 (213.5, 328.5)	0.075
ALB (g/dL)	3.0 ± 0.6	2.6 ± 0.6	2.7 ± 0.5	**0.037**
ALB_cat (decreased)	20.0 (66.6%)	48.0 (81.3%)	23.0 (92.0%)	0.058
PLT (K/μL)	289.0 ± 47.9	304.7 ± 183.3	292.4 ± 170.3	0.906
INR (−)	1.2 (1.2, 1.6)	1.3 (1.2, 1.5)	1.4 (1.2, 1.6)	0.342
PT (sec)	14.0 (12.9, 16.9)	14.5 (13.6, 16.2)	14.8 (13.6, 17.5)	0.471
APTT (sec)	29.7 (27.8, 31.5)	31.7 (28.5, 35.3)	27.1 (29.2, 35.2)	0.155
BUN (mg/dL)	13.0 (8.8, 18.8)	15.0 (9.0, 24.0)	18.0 (13.0, 21.5)	0.173
CR (mg/dL)	0.9 (0.5, 1.1)	0.8 (0.6, 1.5)	0.9 (0.7, 1.1)	0.780
GLU (mg/dL)	110.0 (94.5, 143.0)	134.0 (102.0, 168.0)	111.0 (92.5, 156.5)	0.202
MELD score	8.5 (5.0, 13.8)	10.0 (7.0, 17.0)	12.0 (9.0, 15.0)	0.226
ALBI score	−1.9 ± 0.6	−1.4 ± 0.5	−1.4 ± 0.5	**0.004**
APRI score	0.2 (0.1, 0.4)	0.3 (0.1, 1.2)	0.7 (0.2, 1.9)	**0.001**

*Note:* Bold values indicate significant differences in the results.

Abbreviations: ALB, albumin; ALBI, albumin–bilirubin score; ALP, alkaline phosphatase; ALT, alanine aminotransferase; APRI, Aspartate Aminotransferase to Platelet Ratio Index; APTT, activated partial thromboplastin time; AST, aspartate transaminase; BUN, blood urea nitrogen; cat, category; CR, creatinine; GLU, glucose; HCT, hematocrit; HGB, hemoglobin; INR, international normalized ratio; LDH, lactate dehydrogenase; MELD, model for end–stage liver disease; MONO%, monocyte ratio; NEUT%, neutrophil ratio; PLA, pyogenic liver abscess; PLT, platelet; PT, prothrombin time; RBC, red blood cell; TBIL, total bilirubin; TF, transferrin; TIBC, total iron binding capacity; WBC, white blood cell.

To align with the clinical focus on the “abnormal status” of indicators, iron, ferritin, and ALB were categorized as binary variables in this study, where each was classified into two categories (abnormal or normal) based on the reference range (Table S1). After incorporating the categorical variables into the multinomial regression, the results showed that abnormal ferritin (elevated ferritin in the study) was an independent risk factor for PLA severity (Table 3). Hierarchical regression analyses confirmed that ferritin independently predicts PLA severity, even after accounting for demographic and key liver function indicators (Table 4).

**TABLE 3:** Multivariable regression analysis of clinical characteristics associated with pyogenic liver abscess severity.

	Mild PLA	Moderate PLA	Severe PLA
Iron_cat (decreased)	Reference	2.1 (0.7, 6.8)	13.2 (2.1, 81.2)**
Ferritin_cat (elevated)	Reference	3.9 (1.3, 12.1)*	13.0 (2.9, 59.0)**
ALB_cat (decreased)	Reference	3.1 (0.7, 14.2)	4.1 (0.3, 51.1)

Abbreviations: ALB, albumin; cat, category; PLA, pyogenic liver abscess.

*p < 0.05; **p < 0.01.

**TABLE 4:** Validation of ferritin as an independent risk factor for severe pyogenic liver abscess: Hierarchical regression with covariate adjustment.

Variable	Ferritin's OR (95% CI)			
	Model 1^a^	Model 2^b^	Model 3^c^	Model 4^d^
Mild PLA	Reference	Reference	Reference	Reference
Moderate PLA	5.0 (1.4, 18.3)*	4.5 (1.1, 19.0)*	5.9 (1.2, 29.8)*	5.0 (1.0, 26.2)
Severe PLA	14.3 (2.7, 76.9)**	9.8 (1.5, 69.9)*	12.5 (1.8, 89.0)*	9.8 (1.3, 73.4)*

*Note:* It presented hierarchical regression results focusing on ferritin's odds ratios (ORs) for pyogenic liver abscess (PLA) severity, adjusting for covariates sequentially.

Abbreviations: ALP, alkaline phosphatase; AST, aspartate transaminase; PLA, pyogenic liver abscess; TBIL, total bilirubin.

^a^ Adjusted by gender and age.

^b^ Adjusted by AST.

^c^ Adjusted by ALP.

^d^ Adjusted by TBIL.

*p < 0.05; **p < 0.01.

### 3.4 Verify the Efficiency of Ferritin for Assessing the Severity of PLA

**FIGURE 4:**
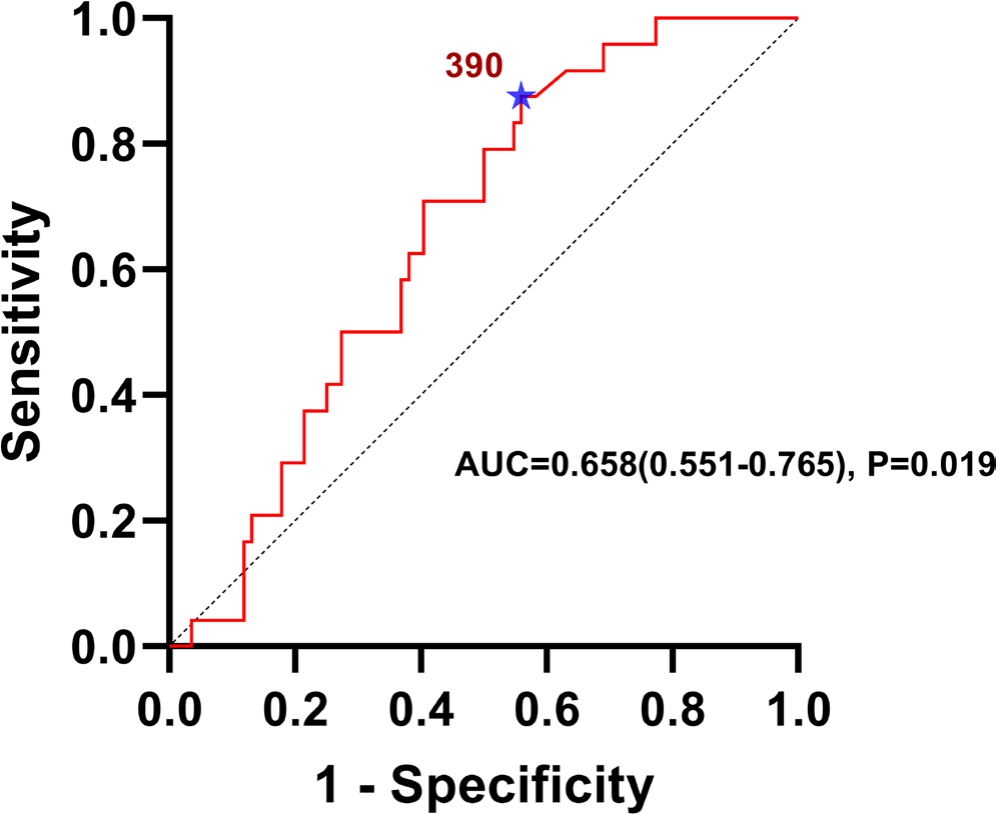
ROC curve of PLA severity assessment by ferritin.

To evaluate the efficiency of identification and diagnosis by ferritin on the severity of PLA, we verified the ability of ferritin by ROC curve analysis (Figure 4), with area under the curve (AUC) of 0.658 (0.551–0.765). To determine the optimal cut‐off value of ferritin, we calculated Youden's Index for each threshold. The results showed that the maximum Youden's Index (0.321) was achieved at a ferritin level of 390 ng/mL, corresponding to a sensitivity of 87.5%.

According to the optimal cut‐off value of ferritin (390 ng/mL), patients with PLA (*n* = 125) were divided into 2 groups: patients with ferritin ≤ 390 ng/mL and patients with ferritin > 390 ng/mL (Table 5). Patients with ferritin > 390 ng/mL had a higher systemic inflammation level (WBC, NEUT%), liver dysfunction (ALT, AST, TBIL, LDH, ALB), iron metabolism disorders (TF, TIBC) and partly biochemical dysfunction (BUN). Besides, MELD score, ALBI score and APRI score were all higher in patients with ferritin > 390 ng/mL. It suggested ferritin acted as a crucial biomarker for screening the severity of PLA.

**TABLE 5:** Comparison of clinical characteristics between the ferritin subgroups in MIMIC‐IV database.

Indicators	Ferritin ≤ 390 ng/mL (*n* = 47)	Ferritin > 390 ng/mL (*n* = 71)	*p*
Gender, male	17.0 (36.1%)	53.0 (74.6%)	**< 0.001**
Age, years	63.0 ± 12.9	61.0 ± 14.1	0.525
Iron (μg/dL)	20.5 (11.8, 34.3)	22.5 (14.3, 33.0)	0.428
TF (mg/dL)	165.2 ± 48.6	134.8 ± 36.9	**< 0.001**
TIBC (μg/dL)	214.8 ± 63.2	175.2 ± 48.0	**< 0.001**
Ferritin (ng/mL)	229.0 (150.0, 297.0)	865.0 (570.0, 237.0)	**< 0.001**
RBC (m/μL)	3.3 (2.8, 3.6)	3.2 (3.0, 3.7)	0.811
HGB (g/dL)	9.1 ± 1.7	9.7 ± 2.0	0.082
HCT (%)	28.4 ± 4.6	29.7 ± 5.7	0.211
WBC (K/μL)	8.6 (6.3, 13.1)	12.2 (9.4, 16.4)	**0.002**
NEUT% (%)	77.3 (67.3, 83.3)	84.0 (75.3, 89.1)	**0.003**
MONO% (%)	6.8 ± 3.7	5.2 ± 3.2	**0.024**
ALT (IU/L)	21.5 (13.0, 41.8)	46.0 (20.0, 68.0)	**0.002**
AST (IU/L)	23.0 (16.3, 34.0)	41.0 (25.0, 70.0)	**< 0.001**
ALP (IU/L)	124.5 (93.5, 342.8)	183.5 (118.0, 265.5)	0.281
TBIL (mg/dL)	0.5 (0.3, 0.8)	0.9 (0.5, 1.7)	**0.002**
LDH (IU/L)	207.5 (152.5, 248.0)	248.0 (213.0, 295.0)	**< 0.001**
ALB (g/dL)	2.9 ± 0.6	2.7 ± 0.5	**0.045**
PLT (K/μL)	316.6 ± 190.8	295.4 ± 168.8	0.526
INR (−)	1.3 (1.2, 1.4)	1.3 (1.2, 1.5)	0.298
PT (sec)	14.5 (13.0, 16.0)	14.7 (13.5, 16.6)	0.235
APTT (sec)	29.7 (28.1, 32.9)	30.4 (27.7, 36.9)	0.817
BUN (mg/dL)	11.0 (8.0, 17.0)	16.5 (10.8, 27.0)	**0.004**
CR (mg/dL)	0.9 (0.5, 1.1)	0.9 (0.7, 1.4)	0.16
GLU (mg/dL)	114.0 (96.0, 157.0)	119.5 (96.0, 155.0)	0.832
MELD score	9.0 (5.0, 13.0)	11.0 (7.5, 17.0)	**0.007**
ALBI score	−1.8 ± 0.5	−1.4 ± 0.5	**0.001**
APRI score	0.2 (0.1, 0.5)	0.3 (0.2, 1.3)	**0.002**

*Note:* Bold values indicate significant differences in the results.

Abbreviations: ALB, albumin; ALBI, albumin–bilirubin score; ALP, alkaline phosphatase; ALT, alanine aminotransferase; APRI, aspartate aminotransferase to platelet ratio index; APTT, activated partial thromboplastin time; AST, aspartate transaminase; BUN, blood urea nitrogen; CR, creatinine; GLU, glucose; HCT, hematocrit; HGB, hemoglobin; INR, international normalized ratio; LDH, lactate dehydrogenase; MELD, model for end–stage liver disease; MONO%, monocyte ratio; NEUT%, neutrophil ratio; PLT, platelet; PT, prothrombin time; RBC, red blood cell; TBIL, total bilirubin; TF, transferrin; TIBC, total iron binding capacity; WBC, white blood cell.

### 3.5 Explore the Efficiency of Ferritin on Recognizing Bacterial Liver Abscess

**FIGURE 5:**
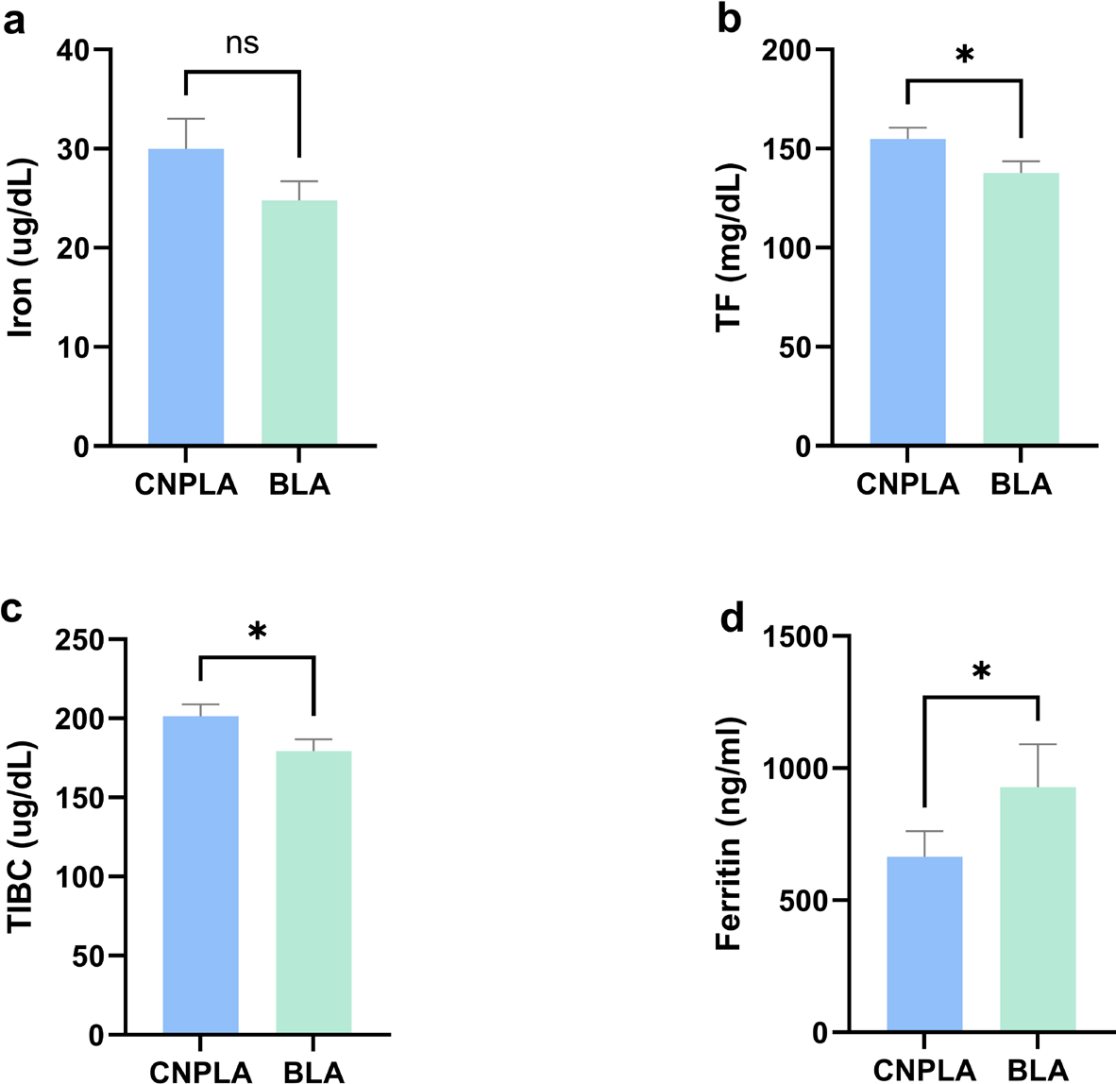
The differential levels of iron metabolism indicators between BLA and CNPLA. (a) The level of iron between BLA and CNPLA; (b) The level of TF between BLA and CNPLA; (c) The level of TIBC between BLA and CNPLA; (d) The level of ferritin between BLA and CNPLA. BLA, bacterial liver abscess; CNPLA, culture negative pyogenic liver abscess; TF, transferrin; TIBC, total iron binding capacity; *p < 0.05; **p < 0.01; ***p < 0.001.

According to the preceding pathogen identification results, patients with BLA account for 49.6% (*n* = 62). Laboratory testing data of these patients presented significant iron metabolism disorders, liver dysfunction and higher systemic levels of inflammation, etc. In other words, the extent of iron metabolism disorders was related to WBC, NEUT%, MONO%, and ALT, AST, ALP, LDH, ALB (Figure S2, Table S19). Compared with CNPLA (50.4%, *n* = 63), these indicators—ferritin, BUN, CR, GLU—were markedly elevated in patients with BLA, while TF, TIBC and ALB were decreased (Figure 5, Table 6). It suggested that ferritin played a pivotal role in identifying and diagnosing BLA. We did further analysis by ROC curve; the result showed that ferritin ≥ 748 ng/mL had a better screening ability on BLA, with a sensitivity of 52.5% and a specificity of 71.2% (Figure S3), which demonstrated that ferritin could act as a key indicator in filtering through BLA from acute PLA.

**TABLE 6:** Comparison of clinical characteristics between BLA and CNPLA.

Indicators	CNPLA (*n* = 63)	BLA (*n* = 62)	*p*
Gender, male	35.0 (55.5%)	39.0 (62.9%)	0.403
Age, years	61.9 ± 12.4	61.6 ± 14.7	0.899
Iron (μg/dL)	23.0 (14.0, 39.8)	22.0 (12.0, 31.5)	0.296
TF (mg/dL)	154.0 (122.0, 189.5)	131.0 (108.5, 165.3)	**0.019**
TIBC (μg/dL)	200.0 (159.0, 246.5)	170.0 (141.3, 215.3)	**0.019**
Ferritin (ng/mL)	349.0 (220.0, 836.0)	770.0 (340.0, 1038.0)	**0.017**
RBC (m/μL)	3.3 (2.9, 3.7)	3.3 (2.8, 3.6)	0.620
HGB (g/dL)	9.6 ± 2.2	9.2 ± 1.7	0.255
HCT (%)	29.7 ± 6.0	28.6 ± 4.8	0.286
WBC (K/μL)	10.1 (6.4, 16.3)	11.9 (8.7, 16.2)	0.084
NEUT% (%)	79.9 (71.5, 85.8)	84.7 (74.9, 90.0)	0.054
MONO% (%)	5.9 ± 3.3	5.5 ± 3.5	0.495
ALT (IU/L)	30.5 (16.8, 56.0)	36.0 (19.3, 67.5)	0.292
AST (IU/L)	31.5 (17.8, 53.3)	33.5 (22.3, 59.5)	0.278
ALP (IU/L)	150.5 (111.5, 289.5)	197.0 (104.8, 275.8)	0.459
TBIL (mg/dL)	0.7 (0.4, 1.3)	0.8 (0.5, 1.5)	0.282
LDH (IU/L)	211.0 (162.0, 251.5)	239.0 (194.0, 325.0)	0.052
ALB (g/dL)	2.9 ± 0.5	2.6 ± 0.5	**0.017**
PLT (K/μL)	288.0 (176.0, 425.0)	284.0 (147.8, 417.5)	0.669
INR (−)	1.3 (1.2, 1.5)	1.3 (1.2, 1.5)	0.545
PT (sec)	14.5 (13.0, 16.4)	14.7 (13.4, 16.6)	0.724
APTT (sec)	30.3 (28.1, 35.1)	30.3 (27.7, 32.9)	0.665
BUN (mg/dL)	13.0 (8.5, 21.5)	16.0 (11.0, 26.0)	**0.026**
CR (mg/dL)	0.8 (0.6, 1.1)	0.9 (0.7, 1.4)	**0.024**
GLU (mg/dL)	111.0 (90.0, 141.5)	137.0 (103.5, 183.0)	**0.003**

*Note:* Bold values indicate significant differences in the results.

Abbreviations: ALB, albumin; ALP, alkaline phosphatase; ALT, alanine aminotransferase; APTT, activated partial thromboplastin time; AST, aspartate transaminase; BLA, bacterial liver abscess; BUN, blood urea nitrogen; CNPLA, culture negative pyogenic liver abscess; CR, creatinine; GLU, glucose; HCT, hematocrit; HGB, hemoglobin; INR, international normalized ratio; LDH, lactate dehydrogenase; MONO%, monocyte ratio; NEUT%, neutrophil ratio; PLT, platelet; PT, prothrombin time; RBC, red blood cell; TBIL, total bilirubin; TF, transferrin; TIBC, total iron binding capacity; WBC, white blood cell.

### 3.6 Validate Iron Metabolism Disorders and the Role of Ferritin in the Clinical Cohort

**FIGURE 6:**
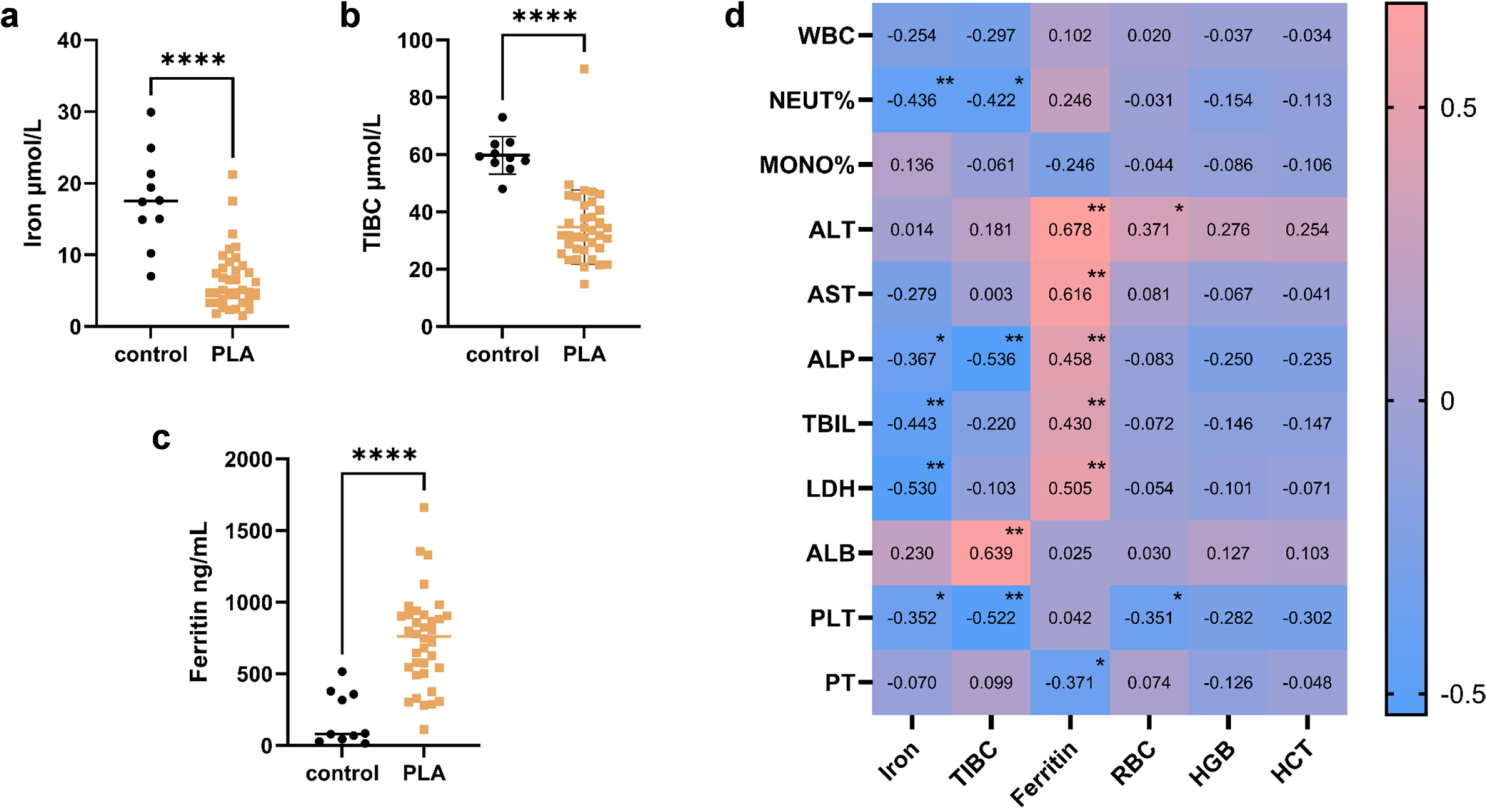
Clinical validation of iron metabolism disorders in PLA: Comparative analysis and correlation analysis. (a–c) Comparison of iron metabolism indicators between the control group and patients with PLA in the clinical validation cohort. (d) Correlation heat map of iron metabolism indicators with liver function and systemic inflammation level in the clinical validation cohort. TIBC, total iron bind capacity; *p < 0.05; **p < 0.01; ****p < 0.0001.

In order to further verify the findings obtained from the MIMIC‐IV database, serum iron, ferritin and TIBC were detected in serum samples of 36 patients and 10 healthy people collected from Peking University Third Hospital. The results showed that the serum iron and TIBC of the patients were significantly reduced, and ferritin was significantly increased (Figure 6a–c), which was consistent with the conclusions drawn from the MIMIC‐IV database. Correlation analysis showed that serum iron, TIBC, and ferritin were significantly correlated with liver function indicators and inflammatory markers to varying degrees, which revealed a significant correlation between iron metabolism disorders in patients with PLA and liver function as well as systemic inflammation levels (Figure 6d).

Based on the levels of ALT and WBC, the patients were stratified into three groups: mild, moderate, and severe. Comparative analysis demonstrated a significant positive association between ferritin levels and disease severity in PLA patients (Table 7), indicating that ferritin served as a reliable biomarker for evaluating the severity of PLA.

**TABLE 7:** Comparison of clinical characteristics between the ferritin subgroups in the clinical cohort.

Indicators	Mild PLA (*n* = 14)	Moderate PLA (*n* = 12)	Severe PLA (*n* = 10)	*p*
Gender, male	7.0 (50.0%)	8.0 (66.6%)	6.0 (60.0%)	0.686
Age, years	67.7 ± 14.7	61.8 ± 14.5	58.1 ± 21.2	0.370
Iron (μg/dL)	6.4 (3.1, 9.2)	6.1 (4.0, 9.6)	4.5 (3.1, 6.8)	0.393
TIBC (μg/dL)	35.3 (26.7, 43.5)	30.0 (22.1, 37.0)	33.1 (29.7, 44.0)	0.415
Ferritin (ng/mL)	560.5 (299.3, 727.0)	787.5 (649.5, 893.3)	907.5 (743.5, 1176.0)	**0.013**
RBC (m/μL)	3.4 ± 0.6	3.5 ± 0.6	4.0 ± 0.9	0.088
HGB (g/dL)	98.1 ± 14.3	104.8 ± 16.6	106.2 ± 19.8	0.442
HCT (%)	0.3 ± 0.1	0.3 ± 0.1	0.3 ± 0.1	0.533
WBC (K/μL)	6.9 ± 1.7	10.3 ± 4.0	17.3 ± 8.6	**< 0.001**
NEUT% (%)	77.0 (67.6, 80.1)	78.8 (73.3, 83.9)	87.9 (78.3, 93.2)	**0.021**
MONO% (%)	6.1 ± 2.7	5.6 ± 2.1	5.3 ± 2.4	0.712
ALT (IU/L)	21.5 (14.0, 35.0)	38.0 (20.3, 48.5)	63.5 (51.5, 98.5)	**< 0.001**
AST (IU/L)	25.5 (18.3, 33.3)	29.0 (23.3, 47.8)	49.0 (37.0, 97.3)	**0.004**
ALP (IU/L)	114.0 (96.0, 146.0)	131.0 (109.3, 220.3)	213.0 (141.0, 291.0)	**0.016**
TBIL (mg/dL)	11.5 (7.6, 21.8)	10.9 (8.7, 16.3)	20.5 (16.7, 25.3)	0.051
LDH (IU/L)	197.0 (150.0, 213.0)	209.0 (160.0, 230.0)	278.5 (215.3, 365.3)	**0.006**
ALB (g/dL)	35.9 ± 4.8	31.5 ± 7.4	31.4 ± 3.5	0.104
PLT (K/μL)	286.0 (236.8, 398.5)	201.5 (143.3, 355.8)	275.5 (64.0, 319.0)	0.270
INR (−)	12.5 ± 1.6	14.1 ± 2.0	13.2 ± 0.8	**0.049**
PT (sec)	1.2 ± 0.2	1.3 ± 0.2	1.2 ± 0.1	**0.039**
APTT (sec)	28.3 ± 8.5	29.5 ± 2.5	31.5 ± 3.2	0.411
BUN (mg/dL)	5.8 ± 2.0	5.5 ± 3.3	8.0 ± 5.7	0.268
CR (mg/dL)	68.0 (51.5, 89.5)	63.5 (54.3, 74.0)	63.0 (42.0, 135.0)	0.907
GLU (mg/dL)	6.3 (6.2, 6.6)	7.4 (6.2, 10.4)	5.7 (5.0, 8.8)	0.181
MELD score	4.0 (0.5, 7.5)	4.0 (3.0, 9.5)	6.0 (2.5, 12.8)	0.377
ALBI score	−2.3 (−2.6, −2.0)	−1.9 (−2.2, −1.6)	−1.9 (−2.1, −1.5)	**0.015**
APRI score	0.2 (0.2, 0.5)	0.4 (0.2, 0.6)	0.7 (0.3, 3.3)	**0.028**

*Note:* Bold values indicate significant differences in the results.

Abbreviations: ALB, albumin; ALBI, albumin–bilirubin score; ALP, alkaline phosphatase; ALT, alanine aminotransferase; APRI, aspartate aminotransferase to platelet ratio index; APTT, activated partial thromboplastin time; AST, aspartate transaminase; BUN, blood urea nitrogen; CR, creatinine; GLU, glucose; HCT, hematocrit; HGB, hemoglobin; INR, international normalized ratio; LDH, lactate dehydrogenase; MELD, model for end–stage liver disease; MONO%, monocyte ratio; NEUT%, neutrophil ratio; PLT, platelet; PT, prothrombin time; RBC, red blood cell; TBIL, total bilirubin; TIBC, total iron bind capacity; WBC, white blood cell.

## 4 Discussion

Pyogenic liver abscess (PLA) is one of the predominant intra‐abdominal infections, the pathogenesis of which is insidious, and the etiology is challenging to elucidate. PLA develops rapidly and exacerbates to invasive liver abscess syndrome, even leading to higher disability and mortality (Yao et al. 2025). A better biomarker should be explored to guide clinical treatment decisions, which is suitable for the early identification of PLA, timely assessment of severity and even contributing to improving the prognosis.

Iron is the most abundant metallic element in the human body, playing an irreplaceable role in basic life activities such as, redox reactions, and electron transfer (Wu et al. 2022). This study found that patients with PLA had significantly altered iron metabolism disorders, manifested by decreased serum iron, TF and TIBC, while ferritin is significantly elevated. More importantly, this metabolic characteristic is closely related to liver function status and systemic inflammation levels, suggesting that iron metabolism disorders may be important participants in the pathological and physiological processes of PLA.

This characteristic change may be the result of a combination of liver dysfunction and host defense mechanisms triggered by infection. As we all know, the liver acts as the central organ regulating iron metabolism (Anderson and Frazer 2017). Our study further confirmed the relationship between liver dysfunction and systematic iron metabolism disorders. On the one hand, the decrease in TF and TIBC was associated with the reduction in ALB and the prolongation of PT, indicating that liver dysfunction in PLA leads to insufficient synthesis of TF, which in turn caused a decrease in TIBC. On the other hand, ferritin is positively correlated with ALT, AST, LDH and TBIL, indicating that it is not only an iron storage molecule, but also a sensitive biomarker of liver parenchymal damage: liver abscess causes hepatocyte necrosis, and the damaged hepatocytes release stored ferritin into the bloodstream. The extent of ferritin elevation corresponds to the severity of hepatocyte necrosis and bilirubin metabolism disorders, which aligns with its nature as an acute‐phase reactive protein. This matches the pattern seen in other liver diseases like non‐alcoholic fatty liver and liver cancer, where impaired liver function is always accompanied by abnormal iron metabolism (Chen et al. 2022; Kowdley et al. 2012; Li et al. 2024; Meier et al. 2020; Suresh et al. 2024; Zhu et al. 2021). However, the difference is that the host's nutritional immune response triggered by infection is another important factor in the iron metabolism disorders of liver abscess (Nairz and Weiss 2020; Shao et al. 2023; Lei et al. 2022). To restrict bacterial iron acquisition, host cells actively transport iron into the cytoplasm and store it in the form of synthesized ferritin. Meanwhile, inflammatory cytokines (such as IL‐6) activate the JAK2‐STAT3 pathway, which induces increased expression of hepcidin in hepatocytes (Ganz and Nemeth 2011; Kernan and Carcillo 2017; Zhang et al. 2021). Hepcidin binds to ferroportin 1 (FPN1), triggering its internalization and degradation (Schmidt 2015). This impairment of cellular iron export reduces serum iron levels while promoting intracellular iron retention, thereby driving ferritin accumulation. In addition, TNF‐α independently enhances ferritin transcription via the NF‐κB pathway, further contributing to elevated ferritin levels (Kou et al. 2013). In this study, it is manifested that systematic inflammatory indicators (WBC, NEUT%) had a negative correlation with serum iron, and had a positive correlation with ferritin. In previous reports, ferritin levels were associated with liver infection diseases (Banchini et al. 2021; Bolarin 1983; Kawamata et al. 2015; Zhou et al. 2020), but we further found that there was a close correlation between ferritin and liver function scores such as MELD, ALBI and APRI; logistic regression analysis confirmed that serum ferritin is an independent factor affecting the severity of PLA—patients with higher ferritin levels showed worse liver dysfunction and higher systemic inflammation levels. The above results indicated that ferritin may serve as a comprehensive biomarker for assessing the severity of PLA. ROC curve analysis further showed the cut‐off 390 ng/mL had a better identification of severity of PLA. At the same time, TF, as the main iron transporter in the blood, may be hijacked by bacteria through its surface transferrin receptors, such as, TF binding protein A, and TF binding protein B (Noinaj et al. 2012). Therefore, the decrease in TF and TIBC during infection (negatively correlated with WBC and NEUT%) is essentially an active defense by the host to reduce the availability of “bacterial iron carriers”. Together with the decrease in serum iron and the increase in ferritin, it forms an iron metabolism network for anti bacterial infections.

However, this adaptive regulation may also pose potential risks. For example, long‐term decrease in serum iron may lead to anemia of inflammation (Ganz 2019; Jiang et al. 2025; Lanser et al. 2021), while excessive increase in ferritin may exacerbate liver damage by promoting oxidative stress (Fernandez‐Rojo et al. 2024), thereby forming a vicious cycle of “infection‐iron metabolism disorders‐organ damage”. This suggests the need for dynamic monitoring of iron metabolism indicators in clinical practice: in the early stage, the adaptive reduction in serum iron and elevation of ferritin can effectively inhibit bacterial growth and have little impact on systemic iron metabolism, so blind iron supplementation should be avoided; when the infection is not controlled promptly, iron homeostasis becomes severely disrupted, leading to severe iron deficiency and pernicious anemia—at this point, targeted iron supplementation is needed to enhance the body's repair ability. This suggests the need for dynamic monitoring of iron metabolism indicators in clinical practice (Nienaber et al. 2023): in the early stage, the adaptive reduction in serum iron and elevation of ferritin can effectively inhibit bacterial growth and have little impact on systemic iron metabolism, so anti—infection should be prioritized. Targeted antibiotics (based on pathogen detection) should be used to control disease progression, as eliminating inflammation is crucial for improving iron metabolism disorders, and blind iron supplementation should be avoided because the elevated ferritin and reduced serum iron at this stage are adaptive antibacterial defenses; supplementation might increase iron availability for bacteria and worsen the infection (Agoro and Mura 2019). Ferritin, serum iron, and inflammatory indicators should be tested every 2–3 days to track iron homeostasis and inflammation. When the infection is not controlled promptly, iron homeostasis becomes severely disrupted, leading to severe iron deficiency and pernicious anemia—at this point, targeted iron supplementation is needed to enhance the body's repair ability. Lactoferrin is a viable option, considering the limitations of oral and intravenous iron: oral iron is associated with frequent gastrointestinal side effects and poor compliance, while intravenous iron, although capable of rapidly increasing hemoglobin, fails to resolve iron utilization disorders and increases long‐term infection risks due to iron overload. Lactoferrin is a multifunctional iron‐binding protein with anti‐inflammatory and antimicrobial properties. Importantly, lactoferrin‐bound iron is not an iron supplement per se, but an immune modulator that affects iron homeostasis through lactoferrin‐dependent signal transduction mechanisms (Zhao et al. 2022). Compared with traditional iron supplements, lactoferrin has better gastrointestinal tolerance and fewer side effects related to high‐dose iron intake. It not only improves blood iron parameters and promotes erythropoiesis more effectively, but also reduces inflammation. There is still a lack of clear guidelines for the treatment of iron deficiency during PLA infection, and there is no unified standard for the dosage, timing, and applicable conditions of iron agents used in clinical practice, which poses challenges to the management of iron metabolism in patients. More targeted clinical trials are needed in the future, with a focus on exploring precise application plans for iron supplements (including new formulations such as lactoferrin) at different stages of the disease, in order to provide a basis for developing evidence‐based guidelines.

Bacterial liver abscess (BLA) is the most common type of PLA (Wendt et al. 2024). Further analysis found that patients with BLA also had iron metabolism disorders, and compared with culture‐negative pyogenic liver abscess (CNPLA), BLA patients had significantly higher levels of ferritin, likely due to stronger bacterial iron demand, which amplifies host iron sequestration. The higher the bacterial load, the stronger the host's iron sequestration response, and the higher the ferritin levels. When pathogen results cannot be obtained immediately, serum ferritin is a fast, convenient, and low‐cost initial screening tool that facilitates early diagnosis and quick treatment decisions for physicians.

This study constructed a cohort of patients with PLA using the MIMIC‐IV database of the USA and originally discovered that iron metabolism disorders are a key factor in the onset and progression of PLA. Among iron metabolism indicators, serum ferritin emerged as a reliable clinical biomarker for reflecting PLA severity and enabling early identification of BLA. These findings were validated in an independent clinical cohort from China. Given the advantages of strong extrapolation value, easy clinical detection, and low cost, ferritin facilitates routine clinical testing and monitoring. However, since patients in the MIMIC‐IV database were predominantly from intensive care units, they may exhibit more severe clinical symptoms, introducing potential selection bias. Additionally, the subgroups had a limited number of patients. This is mainly due to limited retrospective data on iron metabolism markers, as such testing was not routine in PLA's clinical practice previously. These factors suggested that the generalizability of this conclusion might be insufficient, and a larger PLA cohort needs to be built nationwide or even worldwide to pinpoint the reliability and generalizability of PLA influenced by iron metabolism disorders. Furthermore, the optimal ferritin cut‐off value of 390 ng/mL derived from the MIMIC‐IV database was not applicable to the patient cohort of Peking University Third Hospital, which may be attributed to regional disparities in population and differences in measurement methods. However, regardless of Asian or American populations, our study has found that iron metabolism is associated with the severity of pyogenic liver abscess (PLA), and ferritin serves as a key indicator reflecting the severity of PLA. In the future, the role of ferritin in the development of PLA at the molecular mechanism level still needs to be further explored.

## 5 Conclusions

In summary, our study innovatively clarifies the role of iron metabolism disorders (particularly ferritin) in assessing PLA severity and enabling early identification of BLA. Our findings highlight the importance of monitoring iron metabolism indicators in clinical practice, offering new insights for optimizing management, enhancing disease monitoring, and improving prognosis in PLA patients, while the proposed “staged intervention” strategy integrating diet and treatment further provides potential directions for refining clinical rehabilitation pathways.

## Author Contributions

**Zhongyu Han:** methodology (equal), validation (equal), formal analysis (equal), visualization (equal), writing – original draft (equal). **Zhenchao Wu:** conceptualization (equal), methodology (equal), formal analysis (equal), funding acquisition (equal), writing – original draft (equal). **Han Zhou:** data curation (equal), investigation (equal), formal analysis (equal), writing – original draft (equal). **Taikang Yao:** methodology (equal), software (equal), formal analysis (equal), writing – original draft (equal). **Fan Jiang:** methodology (equal), formal analysis (equal), visualization (equal), writing – original draft (equal). **Yingqiu Ying:** formal analysis (equal), supervision (equal), writing – review and editing (equal). **Ming Lu:** formal analysis (equal), supervision (equal), writing – review and editing (equal). **Zihe Zhou:** investigation (equal), formal analysis (equal), writing – original draft (equal). **Zilu Wang:** investigation (equal), formal analysis (equal), writing – original draft (equal). **Ning Shen:** conceptualization (equal), formal analysis (equal), funding acquisition (equal), writing – review and editing (equal). **Jiajia Zheng:** conceptualization (equal), methodology (equal), formal analysis (equal), funding acquisition (equal), writing – review and editing (equal).

## Ethics Statement

The data for this study was obtained from the open‐access database MIMIC‐IV v1.0, which declared that the collection of patient information and the creation of the research resource had been reviewed by the Institutional Review Board at Beth Israel Deaconess Medical Center. The Board granted a waiver of informed consent and approved the data sharing initiative. The cohort from Peking University Third Hospital was a retrospective study with ethical consent waived. Simultaneously, we obtained ethical approval from the Ethics Committee of Peking University Third Hospital (No.: S20241032; M2022382).

## Consent

The authors have nothing to report.

## Conflicts of Interest

The authors declare no conflicts of interest.

## Supporting information


**Figure S1:** The distribution of pathogens in patients with PLA.
**Figure S2:** Correlation heat map of iron metabolism indicators with liver function and systemic inflammation level in patients with BLA.
**Figure S3:** Receiver operating characteristic (ROC) curve analysis of ferritin for detecting bacterial liver abscess (BLA).
**Table S1:** Reference intervals for clinical indicators.
**Table S2:** Difference analysis of indicators among three PLA subgroups before and after imputation using the mean value.
**Table S3:** Difference analysis of the ferritin subgroups before and after imputation using the mean value.
**Table S4:** Correlation between iron metabolism indicators and liver function or systemic inflammation levels in patients with PLA.
**Table S5:** Comparison of clinical characteristics between MELD score ≤ 9 and MELD score > 9 groups in PLA patients.
**Table S6:** Comparison of clinical characteristics between ALBI score ≤ −1.39 and ALBI score > −1.39 groups in PLA patients.
**Table S7:** Comparison of clinical characteristics between APRI score ≤ 2 and APRI score > 2 groups in PLA patients.
**Table S8:** Comparison of clinical characteristics between normal and increased ALT groups in PLA patients.
**Table S9:** Comparison of clinical characteristics between normal and increased AST groups in PLA patients.
**Table S10:** Comparison of clinical characteristics between normal and increased ALP groups in PLA patients.
**Table S11:** Comparison of clinical characteristics between normal and increased LDH groups in PLA patients.
**Table S12:** Comparison of clinical characteristics between normal and increased TBIL groups in PLA patients.
**Table S13:** Comparison of clinical characteristics between normal and decreased ALB groups in PLA patients.
**Table S14:** Comparison of clinical characteristics between normal and increased PT groups in PLA patients.
**Table S15:** Comparison of clinical characteristics between normal and increased WBC groups in PLA patients.
**Table S16:** Comparison of clinical characteristics between normal and increased NEUT% groups in PLA patients.
**Table S17:** Comparison of clinical characteristics between normal and increased MONO% groups in PLA patients.
**Table S18:** Pairwise comparisons of indicators among three PLA subgroups.
**Table S19:** Correlation between iron metabolism indicators and liver function or systemic inflammation levels in patients with BLA.

## Data Availability

The data that support the findings of this study are available from the corresponding author upon reasonable request. The dataset in this study was obtained from MIMIC‐IV v1.0. We had completed the CITI Program course known as Human Research and Data or Specimens Only Research to apply for permission to access the database (Record ID: 40297225).
