# Supplementary material for: Iron Metabolism Disorders Associated With the Severity of Pyogenic Liver Abscess
Source: Food Sci Nutr. 2025 Nov 21;13(11):e71195. doi: 10.1002/fsn3.71195 (PMC12635943; doi:10.1002/fsn3.71195)
Supplement: Supplementary file 1 — Figure S1: The distribution of pathogens in patients with PLA. Figure S2: Correlation heat map of iron metabolism indicators with liver function and systemic inflammation level in patients with BLA. Figure S3: Receiver operating characteristic (ROC) curve analysis of ferritin for detecting bacterial liver abscess (BLA). Table S1: Reference intervals for clinical indicators. Table S2: Difference analysis of indicators among three PLA subgroups before and after imputation using the mean value. Table S3: Difference analysis of the ferritin subgroups before and after imputation using the mean value. Table S4: Correlation between iron metabolism indicators and liver function or systemic inflammation levels in patients with PLA. Table S5: Comparison of clinical characteristics between MELD score ≤ 9 and MELD score > 9 groups in PLA patients. Table S6: Comparison of clinical characteristics between ALBI score ≤ −1.39 and ALBI score > −1.39 groups in PLA patients. Table S7: Comparison of clinical characteristics between APRI score ≤ 2 and APRI score > 2 groups in PLA patients. Table S8: Comparison of clinical characteristics between normal and increased ALT groups in PLA patients. Table S9: Comparison of clinical characteristics between normal and increased AST groups in PLA patients. Table S10: Comparison of clinical characteristics between normal and increased ALP groups in PLA patients. Table S11: Comparison of clinical characteristics between normal and increased LDH groups in PLA patients. Table S12: Comparison of clinical characteristics between normal and increased TBIL groups in PLA patients. Table S13: Comparison of clinical characteristics between normal and decreased ALB groups in PLA patients. Table S14: Comparison of clinical characteristics between normal and increased PT groups in PLA patients. Table S15: Comparison of clinical characteristics between normal and increased WBC groups in PLA patients. Table S16: Comparison of clinical characteristics between no [file FSN3-13-e71195-s001.zip › Supplementary_materials_Iron Metabolism Disorders in Liver Abscess by HZY.docx]

**Supplementary Materials**

**Figure Legends**

**Figure S1. The distribution of pathogens in patients with PLA.** a) Distribution ratio of bacteria sorted by Gram Staining in patients with PLA (10×10 dot plot). b) Bacteria distribution in patients with PLA (pie).

**Figure S2. Correlation heat map of iron metabolism indicators with liver function and systemic inflammation level in patients with BLA.** Note: BLA, bacterial liver abscess; WBC, white blood cell; NEUT%, neutrophil ratio; NEUT#, neutrophil count; MONO%, monocyte ratio; ALT, alanine aminotransferase; AST, aspartate transaminase; ALP, alkaline phosphatase; LDH, lactate dehydrogenase; ALB, albumin; TF, transferrin; TIBC, total iron binding capacity; RBC, red blood cell; HGB, hemoglobin; HCT, hematocrit; *, p < 0.05; **, p < 0.01; ***, p < 0.001.

**Figure S3. Receiver Operating Characteristic (ROC) Curve Analysis of Ferritin for Detecting Bacterial Liver Abscess (BLA).** Note: AUC, area under the curve.

**Table S1. Reference intervals for clinical indicators.**

| Indicators | Units | Reference intervals |
| --- | --- | --- |
| Iron | ug/dL | 45.0-160.0 (male); 30.0-160.0 (female) |
| TF | mg/dL | 200.0-360.0 |
| TIBC | ug/dL | 260.0-470.0 |
| Ferritin | ng/mL | 30.0-400.0 (male); 13.0-150.0 (female) |
| RBC | m/μL | 4.6-6.2 |
| HGB | g/dL | 14.0-18.0 |
| HCT | % | 40.0-52.0 |
| WBC | K/μL | 4.0-11.0 |
| NEUT% | % | 50.0-70.0 |
| MONO% | % | 2.0-11.0 |
| ALT | IU/L | 0.0-40.0 |
| AST | IU/L | 0.0-40.0 |
| ALP | IU/L | 40.0-130.0 |
| TBIL | mg/dL | 0.0-1.5 |
| LDH | IU/L | 94.0-250.0 |
| ALB | g/dL | 3.5-5.2 |
| PLT | K/μL | 150.0-440.0 |
| INR | - | 0.9-1.1 |
| PT | sec | 9.4-12.5 |
| APTT | sec | 25.0-36.5 |
| BUN | mg/dL | 6.0-20.0 |
| CR | mg/dL | 0.4-1.1 |
| GLU | mg/dL | 70.0-100.0 |

Note: TF, transferrin; TIBC, total iron bind capacity; RBC, red blood cell; HGB, hemoglobin; HCT, hematocrit; WBC, white blood cell; NEUT%, neutrophil ratio; MONO%, monocyte ratio; ALT, alanine aminotransferase; AST, aspartate transaminase; ALP, alkaline phosphatase; TBIL, total bilirubin; LDH, lactate dehydrogenase; ALB, albumin; PLT, platelet; INR, international normalized ratio; PT, prothrombin time; APTT, activated partial thromboplastin time; BUN, blood urea nitrogen; CR, creatinine; GLU, glucose.

**Table S2 Difference analysis of indicators among three PLA subgroups before and after imputation using the mean value.**

|  | **Before imputation** | | | | **After imputation** | | | |
| --- | --- | --- | --- | --- | --- | --- | --- | --- |
| **Indicators** | **Mild PLA (n=30)** | **Moderate PLA (n=59)** | **Severe PLA (n=25)** | ***p*-value** | **Mild PLA (n=30)** | **Moderate PLA (n=68)** | **Severe PLA (n=27)** | ***p*-value** |
| Gender, male | 16.0(53.3%) | 34.0(57.6%) | 18.0(72.0%) | 0.336 | 16.0(53.3%) | 29.0(57.3%) | 19(70.3%) | 0.383 |
| Age, years | 62.8±15.5 | 61.2±12.1 | 62.0±16.4 | 0.875 | 62.8±15.5 | 61.4±11.7 | 61.7±15.8 | 0.885 |
| Iron (μg/dL) | 30.0(20.0,43.0) | 19.0(12.5,33.0) | 17.0(11.0,24.0) | **0.009** | 28.5(20.0,41.0) | 22.0(13.0,32.8) | 20.0(11.0,26.0) | **0.017** |
| TF (mg/dL) | 154.9±50.7 | 137.9±43.6 | 139.4±27.5 | 0.326 | 153.8±47.2 | 145.0±45.4 | 141.1±26.5 | 0.457 |
| TIBC (μg/dL) | 201.3±66.0 | 179.2±56.7 | 181.3±35.8 | 0.330 | 199.9±61.4 | 188.6±58.9 | 183.5±34.4 | 0.462 |
| Ferritin (ng/mL) | 310.5(169.8,822.8) | 560.0(314.0,1077.0) | 832.5(510.0,1102.8) | **0.002** | 332.0(171.3,812.3) | 535.0(262.0,941.0) | 795.8(508.0,1102.0) | **0.006** |
| RBC (m/μL) | 3.2(2.8,3.5) | 3.2(2.8,3.7) | 3.4(3.1,3.8) | 0.270 | 3.2±0.5 | 3.3±0.6 | 3.5±0.7 | 0.332 |
| HGB (g/dL) | 8.9(7.7,10.4) | 9.0(7.9,10.3) | 10.1(8.9,11.4) | 0.061 | 8.9(7.7,10.4) | 9.1(7.8,10.4) | 10.0(8.9,11.1) | 0.097 |
| HCT (%) | 28.1(25.0,30.7) | 28.0(25.4,31.3) | 30.7(27.5,34.0) | 0.114 | 28.2(25.0,30.8) | 28.0(25.1,31.5) | 30.5(28.1,33.8) | 0.141 |
| WBC (K/μL) | 7.3±2.2 | 12.5±5.7 | 18.2±6.4 | **<0.001** | 7.0(5.9,9.4) | 11.3(7.5,16.0) | 16.6(12.6,22.4) | **<0.001** |
| NEUT% (%) | 75.0(61.6,83.1) | 81.6(73.9,88.0) | 89.0(81.5,90.7) | **<0.001** | 79.2(63.5,82.3) | 79.2(74.9,86.5) | 86.0(79.2,90.4) | **0.001** |
| MONO% (%) | 6.6±3.7 | 5.4±3.3 | 5.2±3.8 | 0.288 | 5.7(4.2,8.5) | 5.7(4.0,7.0) | 5.6(3.1,6.9) | 0.370 |
| ALT (IU/L) | 19.0(13.8,25.3) | 31.0(17.0,58.0) | 69.0(54.5,93.5) | **<0.001** | 19.0(13.8,25.3) | 36.0(19.3,68.2) | 68.2(56.0,92.0) | **<0.001** |
| AST (IU/L) | 22.0(16.8,29.0) | 33.0(21.0,50.0) | 70.0(38.0,123.5) | **<0.001** | 22.0(16.8,29.0) | 39.0(22.3,61.8) | 70.0(39.0,123.0) | **<0.001** |
| ALP (IU/L) | 103.0(80.8,149.3) | 197.0(136.0,315.0) | 198.0(124.5,368.0) | **<0.001** | 103.0(80.8,149.3) | 236.2(144.0,280.0) | 213.0(125.0,332.0) | **<0.001** |
| TBIL (mg/dL) | 0.4(0.3,0.9) | 0.7(0.4,1.5) | 1.3(0.7,1.9) | **0.001** | 0.4(0.3,1.1) | 1.0(0.4,1.6) | 1.3(0.8,1.7) | **0.001** |
| LDH (IU/L) | 214.0(156.5,252.5) | 220.5(166.8,266.8) | 252.0(213.5,328.5) | 0.075 | 237.5(175.3,249.0) | 249.0(197.8,249.0) | 249.0(228.0,290.0) | 0.105 |
| ALB (g/dL) | 3.0±0.6 | 2.6±0.6 | 2.7±0.5 | **0.037** | 2.9(2.8,3.2) | 2.8(2.3,2.8) | 2.8(2.5,2.9) | **0.021** |
| PLT (K/μL) | 289.0±47.9 | 304.7±183.3 | 292.4±170.3 | 0.906 | 300.0(151.5,385.3) | 283.0(138.0,434.8) | 261.0(157.0,414.0) | 0.999 |
| INR (-) | 1.2(1.2,1.6) | 1.3(1.2,1.5) | 1.4(1.2,1.6) | 0.342 | 1.3(1.2,1.5) | 1.3(1.2,1.4) | 1.4(1.2,1.5) | 0.580 |
| PT (sec) | 14..0(12.9,16.9) | 14.5(13.6,16.2) | 14.8(13.6,17.5) | 0.471 | 14.4(12.9,16.8) | 14.9(13.7,15.8) | 14.7(13.4,16.4) | 0.672 |
| APTT (sec) | 29.7(27.8,31.5) | 31.7(28.5,35.3) | 27.1(29.2,35.2) | 0.155 | 29.9(27.9,32.8) | 32.7(28.6,35.0) | 29.6(27.2,36.4) | 0.143 |
| BUN (mg/dL) | 13.0(8.8,18.8) | 15.0(9.0,24.0) | 18.0(13.0,21.5) | 0.173 | 13.0(8.8,18.8) | 15.0(9.3,23.8) | 18.0(13.0,22.0) | 0.208 |
| CR (mg/dL) | 0.9(0.5,1.1) | 0.8(0.6,1.5) | 0.9(0.7,1.1) | 0.780 | 0.9(0.5,1.1) | 0.8(0.6,1.4) | 0.9(0.7,1.1) | 0.905 |
| GLU (mg/dL) | 110.0(94.5,143.0) | 134.0(102.0,168.0) | 111.0(92.5,156.5) | 0.202 | 110.0(94.5,143.0) | 134.5(102.0,162.3) | 111.0(92.0,152.0) | 0.194 |
| MELD score | 8.5(5.0,13.8) | 10.0(7.0,17.0) | 12.0(9.0,15.0) | 0.226 | 8.5(5.0,13.3) | 10.5(7.3,15.0) | 11.0(9.0,14.0) | 0.129 |
| ALBI score | -1.9±0.6 | -1.4±0.5 | -1.4±0.5 | **0.004** | -1.8±0.6 | -1.5±0.4 | -1.5±0.4 | **0.004** |
| APRI score | 0.2(0.1,0.4) | 0.3(0.1,1.2) | 0.7(0.2,1.9) | **0.001** | 0.2(0.1,0.4) | 0.3(0.2,1.2) | 0.7(0.2,1.8) | **0.001** |

Note: PLA, pyogenic liver abscess; TF, transferrin; TIBC, total iron bind capacity; RBC, red blood cell; HGB, hemoglobin; HCT, hematocrit; WBC, white blood cell; NEUT%, neutrophil ratio; MONO%, monocyte ratio; ALT, alanine aminotransferase; AST, aspartate transaminase; ALP, alkaline phosphatase; TBIL, total bilirubin; LDH, lactate dehydrogenase; ALB, albumin; PLT, platelet; INR, international normalized ratio; PT, prothrombin time; APTT, activated partial thromboplastin time; BUN, blood urea nitrogen; CR, creatinine; GLU, glucose; MELD, Model for End - Stage Liver Disease; ALBI, Albumin - Bilirubin Score; APRI, Aspartate Aminotransferase to Platelet Ratio Index.

**Table S3 Difference analysis of the ferritin subgroups before and after imputation using the mean value.**

|  | **Before imputation** | | | **After imputation** | | |
| --- | --- | --- | --- | --- | --- | --- |
| **Indicators** | **Ferritin≤390ng/mL (n=47)** | **Ferritin>390ng/mL (n=71)** | ***p*-value** | **Ferritin ≤390 ng/mL (n=47)** | **Ferritin >390 ng/mL (n=78)** | ***p*-value** |
| Gender, male | 17.0 (36.1%) | 53.0 (74.6%) | **<0.001** | 17.0 (36.1%) | 57.0 (73%) | **<0.001** |
| Age, years | 63.0±12.9 | 61.0±14.1 | 0.525 | 63.0±12.9 | 61.0±13.9 | 0.530 |
| Iron (μg/dL) | 20.5 (11.8, 34.3) | 22.5 (14.3, 33.0) | 0.428 | 21.0 (12.0, 34.0) | 23.5 (15.0, 33.0) | 0.367 |
| TF (mg/dL) | 165.2±48.6 | 134.8±36.9 | **<0.001** | 164.4±47.7 | 135.4±35.0 | **0.001** |
| TIBC (μg/dL) | 214.8±63.2 | 175.2±48.0 | **<0.001** | 213.7±62.0 | 176.0±45.4 | **0.001** |
| Ferritin (ng/mL) | 229.0 (150.0, 297.0) | 865.0 (570.0, 237.0) | **<0.001** | 229.0 (150.0, 297.0) | 819.0 (610.0, 228.0) | **<0.001** |
| RBC (m/μL) | 3.3 (2.8, 3.6) | 3.2 (3.0, 3.7) | 0.811 | 3.3 (2.8, 3.6) | 3.2 (3.0, 3.6) | 0.716 |
| HGB (g/dL) | 9.1±1.7 | 9.7±2.0 | 0.082 | 9.3 (7.5, 10.4) | 9.2 (8.3, 10.8) | 0.227 |
| HCT (%) | 28.4±4.6 | 29.7±5.7 | 0.211 | 29.0 (24.4, 31.5) | 28.6 (25.9, 32.3) | 0.409 |
| WBC (K/μL) | 8.6 (6.3, 13.1) | 12.2 (9.4, 16.4) | **0.002** | 8.6 (6.3, 13.1) | 12.3 (9.2, 16.6) | **0.002** |
| NEUT% (%) | 77.3 (67.3, 83.3) | 84.0 (75.3,89.1) | **0.003** | 79.2 (72.8, 82.9) | 82.3 (76.4, 88.6) | **0.004** |
| MONO% (%) | 6.8±3.7 | 5.2±3.2 | **0.024** | 5.7 (4.4, 8.2) | 5.5 (3.5, 6.6) | **0.031** |
| ALT (IU/L) | 21.5 (13.0, 41.8) | 46.0 (20.0, 68.0) | **0.002** | 25.0 (13.0, 68.2) | 46.0 (20.8, 68.2) | **0.021** |
| AST (IU/L) | 23.0 (16.3, 34.0) | 41.0 (25.0, 70.0) | **<0.001** | 26.0 (17.0, 47.0) | 45.0 (26.5, 70.6) | **0.001** |
| ALP (IU/L) | 124.5 (93.5, 342.8) | 183.5 (118.0, 265.5) | 0.281 | 160.0 (99.0, 271.0) | 197.0 (125.0, 269.8) | 0.323 |
| TBIL (mg/dL) | 0.5 (0.3, 0.8) | 0.9 (0.5, 1.7) | **0.002** | 0.6 (0.3, 1.3) | 1.1 (0.5, 1.7) | **0.006** |
| LDH (IU/L) | 207.5 (152.5, 248.0) | 248.0 (213.0, 295.0) | **<0.001** | 236.0 (167.0, 249.0) | 249.0 (226.5, 266.5) | **<0.001** |
| ALB (g/dL) | 2.9±0.6 | 2.7±0.5 | **0.045** | 2.9±0.5 | 2.7±0.5 | **0.029** |
| PLT (K/μL) | 316.6±190.8 | 295.4±168.8 | 0.526 | 289.0 (176.0, 461.0) | 281.0 (147.8, 405.3) | 0.519 |
| INR (-) | 1.3 (1.2, 1.4) | 1.3 (1.2, 1.5) | 0.298 | 1.4 (1.2, 1.4) | 1.35 (1.2, 1.5) | 0.558 |
| PT (sec) | 14.5 (13.0, 16.0) | 14.7 (13.5, 16.6) | 0.235 | 14.7 (13.3, 15.6) | 14.9 (13.4, 16.5) | 0.423 |
| APTT (sec) | 29.7 (28.1, 32.9) | 30.4 (27.7, 36.9) | 0.817 | 31.1 (28.5, 35.0) | 31.2 (27.9, 35.0) | 0.931 |
| BUN (mg/dL) | 11.0 (8.0, 17.0) | 16.5 (10.8, 27.0) | **0.004** | 12.0 (8.0, 18.0) | 17.5 (11.0, 27.0) | **0.002** |
| CR (mg/dL) | 0.9 (0.5, 1.1) | 0.9 (0.7, 1.4) | 0.160 | 0.9 (0.5, 1.1) | 0.9 (0.7, 1.4) | 0.240 |
| GLU (mg/dL) | 114.0 (96.0, 157.0) | 119.5 (96.0, 155.0) | 0.832 | 118.0 (100.0, 154.0) | 121.0 (96.0, 156.3) | 0.648 |
| MELD score | 9.0(5.0,13.0) | 11.0(7.5,17.0) | **0.007** | 9.0(6.0,13.0) | 11.5(8.0,16.3) | **0.009** |
| ALBI score | -1.8±0.5 | -1.4±0.5 | **0.001** | -1.8(-2.1,-1.4) | -1.4(-1.7,-1.2) | **<0.001** |
| APRI score | 0.2(0.1,0.5) | 0.3(0.2,1.3) | **0.002** | 0.2(0.1,0.7) | 0.4(0.2,1.3) | **0.008** |

Note: TF, transferrin; TIBC, total iron bind capacity; RBC, red blood cell; HGB, hemoglobin; HCT, hematocrit; WBC, white blood cell; NEUT%, neutrophil ratio; MONO%, monocyte ratio; ALT, alanine aminotransferase; AST, aspartate transaminase; ALP, alkaline phosphatase; TBIL, total bilirubin; LDH, lactate dehydrogenase; ALB, albumin; PLT, platelet; INR, international normalized ratio; PT, prothrombin time; APTT, activated partial thromboplastin time; BUN, blood urea nitrogen; CR, creatinine; GLU, glucose; MELD, Model for End - Stage Liver Disease; ALBI, Albumin - Bilirubin Score; APRI, Aspartate Aminotransferase to Platelet Ratio Index.

**Table S4. Correlation between iron metabolism indicators and liver function or systemic inflammation levels in patients with PLA.**

| Indicators | Iron | | TF | | TIBC | | Ferritin | | RBC | | HGB | | HCT | |
| --- | --- | --- | --- | --- | --- | --- | --- | --- | --- | --- | --- | --- | --- | --- |
|  | r | *p* | r | *p* | r | *p* | r | *p* | r | *p* | r | *p* | r | *p* |
| WBC | -0.246 | **0.007** | -0.266 | **0.004** | -0.266 | **0.004** | 0.275 | **0.003** | 0.010 | 0.908 | 0.060 | 0.508 | 0.073 | 0.417 |
| NEUT% | -0.260 | **0.008** | -0.317 | **0.002** | -0.317 | **0.002** | 0.304 | **0.002** | -0.033 | 0.738 | 0.012 | 0.902 | -0.019 | 0.849 |
| MONO% | -0.048 | 0.630 | 0.222 | **0.029** | 0.222 | **0.029** | -0.200 | **0.045** | 0.147 | 0.131 | 0.053 | 0.590 | 0.054 | 0.581 |
| ALT | -0.081 | 0.399 | 0.208 | **0.034** | 0.208 | **0.034** | 0.349 | **<0.001** | 0.221 | **0.018** | 0.299 | **0.001** | 0.258 | **0.005** |
| AST | -0.080 | 0.407 | 0.111 | 0.260 | 0.111 | 0.260 | 0.371 | **<0.001** | 0.140 | 0.137 | 0.256 | **0.006** | 0.218 | **0.020** |
| ALP | -0.021 | 0.827 | -0.254 | **0.009** | -0.254 | **0.009** | 0.168 | 0.082 | -0.222 | **0.018** | -0.212 | **0.023** | -0.216 | **0.021** |
| TBIL | 0.016 | 0.866 | -0.077 | 0.437 | -0.077 | 0.437 | 0.225 | **0.019** | 0.126 | 0.181 | 0.266 | **0.004** | 0.220 | **0.019** |
| LDH | 0.141 | 0.167 | 0.003 | 0.974 | 0.003 | 0.974 | 0.356 | **<0.001** | 0.027 | 0.791 | 0.138 | 0.168 | 0.145 | 0.147 |
| ALB | 0.109 | 0.277 | 0.689 | **<0.001** | 0.689 | **<0.001** | -0.200 | **0.046** | 0.405 | **<0.001** | 0.381 | **<0.001** | 0.377 | **<0.001** |
| PLT | -0.018 | 0.844 | -0.065 | 0.488 | -0.065 | 0.488 | -0.076 | 0.415 | 0.045 | 0.621 | -0.080 | 0.378 | -0.009 | 0.924 |
| PT | -0.181 | 0.058 | -0.301 | **0.002** | -0.301 | **0.002** | 0.097 | 0.318 | -0.061 | 0.516 | -0.015 | 0.872 | -0.040 | 0.670 |

Note: PLA, pyogenic liver abscess; WBC, white blood cell; NEUT%, neutrophil ratio; MONO%, monocyte ratio; ALT, alanine aminotransferase; AST, aspartate transaminase; ALP, alkaline phosphatase; TBIL, total bilirubin; LDH, lactate dehydrogenase; ALB, albumin; PLT, platelet; PT, prothrombin time; TF, transferrin; TIBC, total iron bind capacity; RBC, red blood cell; HGB, hemoglobin; HCT, hematocrit; r, related coefficient; *p*, *p*-value.

**Table S5. Comparison of Clinical Characteristics Between MELD score≤9 and MELD score>9 Groups in PLA Patients.**

| Indicators | PLA patients with MELD score≤9  (n=50) | PLA patients with MELD score>9  (n=59) | *p*-value |
| --- | --- | --- | --- |
| Gender, male | 27.0(54%) | 36.0(61%) | 0.460 |
| Age, years | 62.5(55.0,71.0) | 61.0(50.0,70.0) | 0.477 |
| Iron (μg/dL) | 17.5(11.0,30.3) | 22.0(16.0,35.0) | 0.055 |
| TF (mg/dL) | 145.3±39.1 | 138.4±46.7 | 0.429 |
| TIBC (μg/dL) | 188.9±50.9 | 180.0±60.7 | 0.433 |
| Ferritin (ng/mL) | 402.5(269.8,834.0) | 798.0(333.0,1234.0) | **0.035** |
| RBC (m/μL) | 3.3(3.1,3.6) | 3.1(2.7,3.6) | 0.101 |
| HGB (g/dL) | 9.1(8.3,10.3) | 9.2(7.6,10.7) | 0.959 |
| HCT (%) | 28.3(25.9,31.0) | 28.7(23.2,32.1) | 0.597 |
| WBC (K/μL) | 11.1(7.5,16.3) | 12.3(6.9,16.6) | 0.724 |
| NEUT% (%) | 81.0(73.9,87.5) | 84.0(74.2,88.4) | 0.486 |
| MONO% (%) | 5.2±3.3 | 6.1±3.7 | 0.242 |
| ALT (IU/L) | 26.5(16.0,55.3) | 40.0(20.8,69.3) | 0.070 |
| AST (IU/L) | 26.5(18.5,44.5) | 40.5(24.3,73.5) | **0.011** |
| ALP (IU/L) | 142.5(91.5,274.3) | 197.5(133.3,309.8) | **0.021** |
| TBIL (mg/dL) | 0.4(0.3,0.7) | 1.3(0.6,2.1) | **<0.001** |
| LDH (IU/L) | 212.5(165.3,269.0) | 233.0(194.0,278.0) | 0.248 |
| ALB (g/dL) | 2.8±0.5 | 2.7±0.6 | 0.333 |
| PLT (K/μL) | 323.0(242.3,438.0) | 220.0(117.0,342.0) | **0.009** |
| INR (-) | 1.3(1.2,1.4) | 1.4(1.3,1.7) | **<0.001** |
| PT (sec) | 14.0(13.0,15.2) | 15.6(13.8,18.5) | **<0.001** |
| APTT (sec) | 29.1(27.8,32.0) | 31.6(28.7,37.4) | **0.007** |
| BUN (mg/dL) | 13.0(9.0,18.0) | 18.0(11.0,32.0) | **0.003** |
| CR (mg/dL) | 0.8(0.6,0.9) | 1.0(0.7,1.8) | **<0.001** |
| GLU (mg/dL) | 116.5(92.0,146.0) | 125.0(102.0,169.0) | 0.166 |

Note: TF, transferrin; TIBC, total iron bind capacity; RBC, red blood cell; HGB, hemoglobin; HCT, hematocrit; WBC, white blood cell; NEUT%, neutrophil ratio; MONO%, monocyte ratio; ALT, alanine aminotransferase; AST, aspartate transaminase; ALP, alkaline phosphatase; TBIL, total bilirubin; LDH, lactate dehydrogenase; ALB, albumin; PLT, platelet; INR, international normalized ratio; PT, prothrombin time; APTT, activated partial thromboplastin time; BUN, blood urea nitrogen; CR, creatinine; GLU, glucose.

**Table S6. Comparison of Clinical Characteristics Between ALBI score≤-1.39 and ALBI score>-1.39 Groups in PLA Patients.**

| Indicators | PLA patients with ALBI score≤-1.39  (n=61) | PLA patients with ALBI score>-1.39  (n=41) | *p*-value |
| --- | --- | --- | --- |
| Gender, male | 35.0(57.3%) | 24.0(58.5%） | 0.907 |
| Age, years | 63.5±13.8 | 58.2±14.4 | 0.065 |
| Iron (μg/dL) | 21.0(13.5,40.0) | 20.0(12.0,28.0) | 0.275 |
| TF (mg/dL) | 156.7±41.5 | 116.6±33.1 | **<0.001** |
| TIBC (μg/dL) | 203.7±54.0 | 151.6±43.0 | **<0.001** |
| Ferritin (ng/mL) | 396.0(254.5,869.5) | 805.5(523.8,195.3) | **0.003** |
| RBC (m/μL) | 3.4(3.1,3.7) | 3.0(2.7,3.4) | **0.010** |
| HGB (g/dL) | 9.5(8.3,10.6) | 8.9(7.6,9.9) | 0.080 |
| HCT (%) | 29.2(25.7,32.3) | 26.7(23.6,30.7) | **0.043** |
| WBC (K/μL) | 10.4(6.9,15.7) | 12.4(10.0,16.7) | 0.217 |
| NEUT% (%) | 80.9(72.3,87.1) | 86.0(77.0,90.4) | 0.077 |
| MONO% (%) | 6.1±3.6 | 5.1±3.6 | 0.216 |
| ALT (IU/L) | 35.5(19.0,70.5) | 31.0(19.5,53.0) | 0.589 |
| AST (IU/L) | 30.5(19.3,47.8) | 40.0(24.0,59.5) | 0.196 |
| ALP (IU/L) | 128.0(93.5,275.5) | 225.0(146.5,369.5) | **0.015** |
| TBIL (mg/dL) | 0.5(0.3,1.1) | 1.3(0.8,2.5) | **<0.001** |
| LDH (IU/L) | 215.0(168.3,261.5) | 239.5(188.8,326.3) | 0.217 |
| ALB (g/dL) | 3.0±0.5 | 2.4±0.4 | **<0.001** |
| PLT (K/μL) | 319.4±76.7 | 243.8±165.8 | **0.032** |
| INR (-) | 1.3(1.2,1.4) | 1.4(1.2,1.7) | **0.034** |
| PT (sec) | 14.4(13.3,15.8) | 15.5(13.7,18.6) | **0.036** |
| APTT (sec) | 29.6(27.6,33.0) | 31.2(28.3,37.1) | 0.102 |
| BUN (mg/dL) | 14.0(10.0,21.5) | 18.0(10.5,28.5) | 0.182 |
| CR (mg/dL) | 0.9(0.7,1.2) | 0.9(0.7,1.6) | 0.522 |
| GLU (mg/dL) | 123.0(102.0,155.5) | 125.0(101.0,189.0) | 0.501 |

Note: TF, transferrin; TIBC, total iron bind capacity; RBC, red blood cell; HGB, hemoglobin; HCT, hematocrit; WBC, white blood cell; NEUT%, neutrophil ratio; MONO%, monocyte ratio; ALT, alanine aminotransferase; AST, aspartate transaminase; ALP, alkaline phosphatase; TBIL, total bilirubin; LDH, lactate dehydrogenase; ALB, albumin; PLT, platelet; INR, international normalized ratio; PT, prothrombin time; APTT, activated partial thromboplastin time; BUN, blood urea nitrogen; CR, creatinine; GLU, glucose.

**Table S7. Comparison of Clinical Characteristics Between APRI score≤2 and APRI score>2 Groups in PLA Patients.**

| Indicators | PLA patients with APRI score≤2  (n=99) | PLA patients with APRI score>2  (n=15) | *p*-value |
| --- | --- | --- | --- |
| Gender, male | 63.0(63.6%) | 5.0(33.3%) | **0.026** |
| Age, years | 61.6±13.1 | 63.5±19.2 | 0.716 |
| Iron (μg/dL) | 22.0(13.0,33.5) | 17.0(12.0,30.0) | 0.442 |
| TF (mg/dL) | 141.3±43.5 | 152.2±36.0 | 0.429 |
| TIBC (μg/dL) | 183.8±56.5 | 197.9±46.8 | 0.427 |
| Ferritin (ng/mL) | 519.5(273.5,937.5) | 834.5(718.0,1898.0) | **0.016** |
| RBC (m/μL) | 3.3(2.8,3.6) | 3.3(3.0,4.1) | 0.213 |
| HGB (g/dL) | 9.0(7.8,10.3) | 10.4(9.2,12.7) | **0.006** |
| HCT (%) | 28.0(25.2,31.5) | 31.3(28.6,36.7) | **0.016** |
| WBC (K/μL) | 11.4(7.2,16.3) | 11.1(9.4,16.9) | 0.734 |
| NEUT% (%) | 81.5(72.8,87.0) | 90.4(75.9,93.3) | **0.024** |
| MONO% (%) | 5.6(3.9,7.9) | 4.0(1.7,6.9) | 0.181 |
| ALT (IU/L) | 27.0(17.0,53.0) | 142.0(64.0,372.0) | **<0.001** |
| AST (IU/L) | 29.0(19.0,46.0) | 151.0(85.0,305.0) | **<0.001** |
| ALP (IU/L) | 158.0(99.0,277.0) | 189.0(141.0,404.0) | 0.239 |
| TBIL (mg/dL) | 0.6(0.4,1.3) | 1.3(0.8,4.5) | **0.002** |
| LDH (IU/L) | 219.0(166.8,260.0) | 252.0(222.0,397.0) | **0.019** |
| ALB (g/dL) | 2.7±0.5 | 2.9±0.7 | 0.220 |
| PLT (K/μL) | 323.0±166.0 | 132.1±87.8 | **<0.001** |
| INR (-) | 1.3(1.2,1.5) | 1.3(1.1,1.7) | 0.584 |
| PT (sec) | 14.7(13.5,16.3) | 13.8(12.1,17.8) | 0.398 |
| APTT (sec) | 30.0(28.1,33.3) | 31.6(27.6,39.3) | 0.458 |
| BUN (mg/dL) | 14.0(10.0,22.0) | 19.0(10.0,27.0) | 0.155 |
| CR (mg/dL) | 0.9(0.6,1.2) | 0.8(0.6,1.3) | 0.956 |
| GLU (mg/dL) | 118.0(96.0,155.0) | 143.0(101.0,189.0) | 0.129 |

Note: TF, transferrin; TIBC, total iron bind capacity; RBC, red blood cell; HGB, hemoglobin; HCT, hematocrit; WBC, white blood cell; NEUT%, neutrophil ratio; MONO%, monocyte ratio; ALT, alanine aminotransferase; AST, aspartate transaminase; ALP, alkaline phosphatase; TBIL, total bilirubin; LDH, lactate dehydrogenase; ALB, albumin; PLT, platelet; INR, international normalized ratio; PT, prothrombin time; APTT, activated partial thromboplastin time; BUN, blood urea nitrogen; CR, creatinine; GLU, glucose.

**Table S8. Comparison of Clinical Characteristics Between Normal and Increased ALT Groups in PLA Patients.**

| Indicators | PLA patients with normal ALT  （n=67） | PLA patients  with increased ALT  （n=47） | *p*-value |
| --- | --- | --- | --- |
| Gender, male | 36.0 (53.7%) | 32.0 (68%) | 0.124 |
| Age, years | 62.0±13.7 | 62.0±14.4 | 0.899 |
| Iron (μg/dL) | 24.0 (13.0,38.0) | 18.0 (12.0,27.0) | 0.128 |
| TF (mg/dL) | 138.6±46.0 | 147.5±38.0 | 0.295 |
| TIBC (μg/dL) | 180.2±59.7 | 191.9±49.4 | 0.289 |
| Ferritin (ng/mL) | 426.0 (231.3,877.3) | 780.5 (405.8,268.0) | **0.010** |
| RBC (m/μL) | 3.2 (2.8,3.5) | 3.3 (3.0,3.9) | 0.078 |
| HGB (g/dL) | 9.1±1.7 | 9.9±2.2 | **0.030** |
| HCT (%) | 28.4±4.9 | 30.2±6.3 | 0.103 |
| WBC (K/μL) | 12.0±5.4 | 12.9±7.6 | 0.504 |
| NEUT% (%) | 81.3 (74.2,86.0) | 84.0 (73.2,90.4) | 0.120 |
| MONO% (%) | 5.0 (3.6,7.0) | 5.8 (3.1,8.2) | 0.510 |
| ALT (IU/L) | 20.0 (13.0,27.0) | 68.0 (55.0,95.0) | **<0.001** |
| AST (IU/L) | 24.0 (17.0,34.0) | 57.0 (37.0,121.0) | **<0.001** |
| ALP (IU/L) | 153.0 (92.0,269.0) | 197.0 (131.0,366.0) | **0.023** |
| TBIL (mg/dL) | 0.6 (0.4,1.3) | 1.0 (0.5,2.1) | **0.010** |
| LDH (IU/L) | 214.0 (171.5,265.5) | 229.0 (188.5,291.5) | 0.599 |
| ALB (g/dL) | 2.6±0.5 | 2.9±0.5 | **0.049** |
| PLT (K/μL) | 322.0 (210.0,447.0) | 220.0 (117.0,384.0) | **0.008** |
| INR (-) | 1.3 (1.2,1.5) | 1.4 (1.2,1.5) | 0.222 |
| PT (sec) | 14.5 (13.0,16.6) | 14.7 (13.6,16.2) | 0.456 |
| APTT (sec) | 29.9 (28.1,32.6) | 30.7 (27.6,37.4) | 0.457 |
| BUN (mg/dL) | 14.0 (9.0,24.0) | 16.0 (10.0,21.0) | 0.370 |
| CR (mg/dL) | 0.9 (0.6,1.4) | 0.8 (0.7,1.1) | 0.929 |
| GLU (mg/dL) | 118.0 (96.0,169.0) | 129.0 (101.0,155.0) | 0.890 |

Note: TF, transferrin; TIBC, total iron bind capacity; RBC, red blood cell; HGB, hemoglobin; HCT, hematocrit; WBC, white blood cell; NEUT%, neutrophil ratio; MONO%, monocyte ratio; ALT, alanine aminotransferase; AST, aspartate transaminase; ALP, alkaline phosphatase; TBIL, total bilirubin; LDH, lactate dehydrogenase; ALB, albumin; PLT, platelet; INR, international normalized ratio; PT, prothrombin time; APTT, activated partial thromboplastin time; BUN, blood urea nitrogen; CR, creatinine; GLU, glucose.

**Table S9. Comparison of Clinical Characteristics Between Normal and Increased AST Groups in PLA Patients.**

| Indicators | PLA patients with normal AST  （n=70） | PLA patients with increased AST  （n=44） | *p*-value |
| --- | --- | --- | --- |
| Gender, male | 39.0 (55.7%) | 29.0 (65.9%) | 0.280 |
| Age, years | 61.0±13.7 | 63.0±14.5 | 0.527 |
| Iron (μg/dL) | 24.0 (12.0,36.5) | 19.0 (13.0,27.0) | 0.223 |
| TF (mg/dL) | 143.5±42.2 | 140.7±44.1 | 0.743 |
| TIBC (μg/dL) | 186.6±54.9 | 182.9±57.3 | 0.747 |
| Ferritin (ng/mL) | 366.5 (254.3,870.0) | 807.0 (523.8,1418.5) | **<0.001** |
| RBC (m/μL) | 3.3 (2.9,3.6) | 3.3 (2.9,3.7) | 0.993 |
| HGB (g/dL) | 9.2±1.7 | 9.9±2.3 | 0.057 |
| HCT (%) | 28.2±4.8 | 30.1±6.4 | 0.163 |
| WBC (K/μL) | 11.2 (6.8,16.2) | 12 (8.6,16.8) | 0.311 |
| NEUT% (%) | 80.6 (71.9,85.9) | 87.0 (76.1,90.6) | **0.002** |
| MONO% (%) | 6.2±3.7 | 5.0±3.2 | 0.119 |
| ALT (IU/L) | 20.0 (13.8,35.3) | 66.5 (42.5,116.8) | **<0.001** |
| AST (IU/L) | 23.0 (16.8,30.3) | 63.5 (49.3,122.5) | **<0.001** |
| ALP (IU/L) | 150.5 (92.8,262.5) | 202.0 (132.3,406.3) | **0.011** |
| TBIL (mg/dL) | 0.5 (0.3,1.1) | 1.3 (0.6,2.7) | **<0.001** |
| LDH (IU/L) | 213.0 (168.0,260.0) | 240.5 (193.0,294.3) | 0.110 |
| ALB (g/dL) | 2.7±0.5 | 2.8±0.6 | 0.616 |
| PLT (K/μL) | 324.5 (218.3,458.0) | 191.0 (110.0,304.5) | **<0.001** |
| INR (-) | 1.3 (1.2,1.5) | 1.3 (1.2,1.6) | 0.569 |
| PT (sec) | 14.7 (13.4,16.2) | 14.7 (13.2,17.7) | 0.688 |
| APTT (sec) | 29.7 (27.9,32.3) | 32.0 (28.5,41.0) | 0.060 |
| BUN (mg/dL) | 12.5 (9.0,21.0) | 18.5 (11.5,26.5) | **0.008** |
| CR (mg/dL) | 0.9 (0.6,1.1) | 0.8 (0.6,1.5) | 0.561 |
| GLU (mg/dL) | 116.5 (99.0,145.0) | 132.0 (96.0,170.0) | 0.397 |

Note: TF, transferrin; TIBC, total iron bind capacity; RBC, red blood cell; HGB, hemoglobin; HCT, hematocrit; WBC, white blood cell; NEUT%, neutrophil ratio; MONO%, monocyte ratio; ALT, alanine aminotransferase; AST, aspartate transaminase; ALP, alkaline phosphatase; TBIL, total bilirubin; LDH, lactate dehydrogenase; ALB, albumin; PLT, platelet; INR, international normalized ratio; PT, prothrombin time; APTT, activated partial thromboplastin time; BUN, blood urea nitrogen; CR, creatinine; GLU, glucose.

**Table S10. Comparison of Clinical Characteristics Between Normal and Increased ALP Groups in PLA Patients.**

| Indicators | PLA patients with normal ALP  (n=41) | PLA patients with increased ALP  (n=73) | *p*-value |
| --- | --- | --- | --- |
| Gender, male | 24.0 (58.5%) | 44.0 (60.2%) | 0.856 |
| Age, years | 63.0±16.0 | 61.0±12.8 | 0.641 |
| Iron (μg/dL) | 22.0 (15.0,39.0) | 21.0 (12.0,30.5) | 0.213 |
| TF (mg/dL) | 161.8±39.9 | 130.4±40.2 | **<0.001** |
| TIBC (μg/dL) | 210.3±51.9 | 169.6±52.2 | **<0.001** |
| Ferritin (ng/mL) | 349.0 (224.5,808.5) | 770.0 (340.0,1103.0) | **0.005** |
| RBC (m/μL) | 3.4 (3.1,3.9) | 3.1 (2.8,3.6) | **0.026** |
| HGB (g/dL) | 9.9±2.1 | 9.2±1.9 | 0.050 |
| HCT (%) | 30.6±5.5 | 28.3±5.4 | **0.035** |
| WBC (K/μL) | 10.1 (6.8,13.2) | 12.6 (8.6,17.1) | **0.013** |
| NEUT% (%) | 81.0 (72.7,86.5) | 83.0 (75.1,90.0) | 0.220 |
| MONO% (%) | 5.7±3.5 | 5.7±3.5 | 0.980 |
| ALT (IU/L) | 20.0 (17.5,43.0) | 40.0 (19.0,66.5) | **0.029** |
| AST (IU/L) | 25.0 (18.5,40.5) | 37.0 (24.0,64.0) | **0.018** |
| ALP (IU/L) | 92.0 (78.5,114.0) | 248.0 (171.0,408.0) | **<0.001** |
| TBIL (mg/dL) | 0.5 (0.3,1.1) | 1.0 (0.5,1.8) | **<0.001** |
| LDH (IU/L) | 237.0 (180.3,300.3) | 222.0 (172.0,266.0) | 0.391 |
| ALB (g/dL) | 2.9±0.5 | 2.6±0.5 | **0.005** |
| PLT (K/μL) | 318.4±171.2 | 286.3±170.1 | 0.336 |
| INR (-) | 1.4 (1.2,1.5) | 1.3 (1.2,1.6) | 0.392 |
| PT (sec) | 14.9 (13.2,16.3) | 14.7 (13.7,17.2) | 0.463 |
| APTT (sec) | 28.8 (27.7,31.3) | 31.3 (28.2,27.3) | **0.013** |
| BUN (mg/dL) | 14.0 (9.0,20.5) | 15.0 (10.0,24.5) | 0.253 |
| CR (mg/dL) | 0.8 (0.7,1.1) | 0.9 (0.6,1.4) | 0.446 |
| GLU (mg/dL) | 123.0 (101.5,162.5) | 119.0 (93.0,158.5) | 0.599 |

Note: TF, transferrin; TIBC, total iron bind capacity; RBC, red blood cell; HGB, hemoglobin; HCT, hematocrit; WBC, white blood cell; NEUT%, neutrophil ratio; MONO%, monocyte ratio; ALT, alanine aminotransferase; AST, aspartate transaminase; ALP, alkaline phosphatase; TBIL, total bilirubin; LDH, lactate dehydrogenase; ALB, albumin; PLT, platelet; INR, international normalized ratio; PT, prothrombin time; APTT, activated partial thromboplastin time; BUN, blood urea nitrogen; CR, creatinine; GLU, glucose.

**Table S11. Comparison of Clinical Characteristics Between Normal and Increased LDH Groups in PLA Patients.**

| Indicators | PLA patients with normal LDH  (n=68) | PLA patients with increased LDH  (n=34) | *p*-value |
| --- | --- | --- | --- |
| Gender, male | 38.0 (55.8%) | 20.0 (58.8%) | 0.777 |
| Age, years | 61.0±13.1 | 64.0±15.1 | 0.434 |
| Iron (μg/dL) | 20.0 (12.8,32.3) | 24.0 (15.3,33.8) | 0.359 |
| TF (mg/dL) | 145.3±46.4 | 137.7±37.2 | 0.428 |
| TIBC (μg/dL) | 188.9±60.4 | 179.1±48.3 | 0.427 |
| Ferritin (ng/mL) | 417.0 (228.0,804.0) | 847.5 (557.8,1265.8) | **0.001** |
| RBC (m/μL) | 3.2 (3.0,3.6) | 3.3 (2.8,3.5) | 0.859 |
| HGB (g/dL) | 9.2 (7.9,10.3) | 9.1 (7.6,11.0) | 0.657 |
| HCT (%) | 28.7±5.2 | 29.5±6.4 | 0.516 |
| WBC (K/μL) | 10.2 (6.8,14.7) | 14.1 (10.4,18.0) | **0.003** |
| NEUT% (%) | 79.5 (72.1,87.0) | 85.7 (78.2,89.9) | **0.027** |
| MONO% (%) | 5.9±3.5 | 5.4±3.7 | 0.464 |
| ALT (IU/L) | 36.0 (16.0,65.5) | 36.0 (20.0,70.0) | 0.309 |
| AST (IU/L) | 30.5 (19.0,53.0) | 41.0 (23.5,90.0) | 0.072 |
| ALP (IU/L) | 171.0 (117.5,331.0) | 136.0 (90.5,263.0) | 0.357 |
| TBIL (mg/dL) | 0.7 (0.4,1.3) | 1.1 (0.6,1.8) | 0.079 |
| LDH (IU/L) | 194.0 (158.3,227.8) | 330.5 (267.5,444.0) | **<0.001** |
| ALB (g/dL) | 2.7±0.6 | 2.7±0.6 | 0.827 |
| PLT (K/μL) | 263.0 (119.3,454.5) | 286.5 (155.3,354.8) | 0.826 |
| INR (-) | 1.3 (1.2,1.6) | 1.4 (1.2,1.5) | 0.745 |
| PT (sec) | 14.7 (13.2,17.1) | 14.7 (13.4,16.2) | 0.970 |
| APTT (sec) | 31.2 (28.4,35.7) | 29.6 (27.6,32.7) | 0.136 |
| BUN (mg/dL) | 13.0 (10.0,21.0) | 18.0 (13.0,30.5) | **0.034** |
| CR (mg/dL) | 0.8 (0.5,1.2) | 0.9 (0.7,1.5) | 0.194 |
| GLU (mg/dL) | 115.0 (92.0,155.0) | 132.0 (104.0,194.5) | 0.070 |

Note: TF, transferrin; TIBC, total iron bind capacity; RBC, red blood cell; HGB, hemoglobin; HCT, hematocrit; WBC, white blood cell; NEUT%, neutrophil ratio; MONO%, monocyte ratio; ALT, alanine aminotransferase; AST, aspartate transaminase; ALP, alkaline phosphatase; TBIL, total bilirubin; LDH, lactate dehydrogenase; ALB, albumin; PLT, platelet; INR, international normalized ratio; PT, prothrombin time; APTT, activated partial thromboplastin time; BUN, blood urea nitrogen; CR, creatinine; GLU, glucose.

**Table S12. Comparison of Clinical Characteristics Between Normal and Increased TBIL Groups in PLA Patients.**

| Indicators | PLA patients with normal TBIL  (n=89) | PLA patients with increased TBIL  (n=25) | *p*-value |
| --- | --- | --- | --- |
| Gender, male | 53.0 (59.5%) | 15.0 (69%) | 0.968 |
| Age, years | 62.0±13.4 | 60.0±16.0 | 0.60 |
| Iron (μg/dL) | 20.5 (12.3,33.0) | 23.0 (15.8,28.8) | 0.619 |
| TF (mg/dL) | 144.9±43.7 | 134.4±39.8 | 0.330 |
| TIBC (μg/dL) | 188.3±56.9 | 174.9±51.6 | 0.335 |
| Ferritin (ng/mL) | 507.0 (278.5,958.0) | 770.0 (516.0,1038.0) | 0.154 |
| RBC (m/μL) | 3.3 (2.9,3.6) | 3.2 (2.9,3.9) | 0.975 |
| HGB (g/dL) | 9.1 (7.8,10.4) | 9.8 (8.6,11.5) | 0.164 |
| HCT (%) | 28.0 (25.5,31.6) | 29.9 (26.0,34.6) | 0.317 |
| WBC (K/μL) | 11.2 (7.1,16.4) | 11.7 (8.2,14.8) | 0.948 |
| NEUT% (%) | 81.8 (73.5,87.2) | 83.0 (74.9,90.4) | 0.369 |
| MONO% (%) | 5.7 (3.8,7.6) | 5.0 (2.6,6.9) | 0.585 |
| ALT (IU/L) | 29.5 (17.0,57.5) | 50.0 (26.5,71.5) | **0.029** |
| AST (IU/L) | 29.0 (19.0,46.8) | 61.0 (35.0,114.0) | **<0.001** |
| ALP (IU/L) | 155.0 (99.0,280.0) | 206.0 (137.0,300.5) | 0.120 |
| TBIL (mg/dL) | 0.6 (0.4,1.0) | 2.8 (2.0,5.2) | **<0.001** |
| LDH (IU/L) | 214.0 (166.5,261.5) | 247.0 (233.3,347.3) | **0.013** |
| ALB (g/dL) | 2.7±0.5 | 2.7±0.6 | 0.938 |
| PLT (K/μL) | 317.7±174.3 | 209.6±132.9 | **0.002** |
| INR (-) | 1.3 (1.2,1.5) | 1.4(1.2,1.7) | 0.211 |
| PT (sec) | 14.7 (13.3,16.2) | 15.0 (13.8,18.4) | 0.237 |
| APTT (sec) | 30.2 (28.1,33.2) | 30.4 (27.8,37.5) | 0.874 |
| BUN (mg/dL) | 14.0 (10.0,21.5) | 17.0 (9.0,32.5) | 0.549 |
| CR (mg/dL) | 0.8 (0.6,1.2) | 0.9 (0.7,1.4) | 0.439 |
| GLU (mg/dL) | 121.0 (96.0,158.5) | 114.0 (98.5,149.0) | 0.689 |

Note: TF, transferrin; TIBC, total iron bind capacity; RBC, red blood cell; HGB, hemoglobin; HCT, hematocrit; WBC, white blood cell; NEUT%, neutrophil ratio; MONO%, monocyte ratio; ALT, alanine aminotransferase; AST, aspartate transaminase; ALP, alkaline phosphatase; TBIL, total bilirubin; LDH, lactate dehydrogenase; ALB, albumin; PLT, platelet; INR, international normalized ratio; PT, prothrombin time; APTT, activated partial thromboplastin time; BUN, blood urea nitrogen; CR, creatinine; GLU, glucose.

**Table S13. Comparison of Clinical Characteristics Between Normal and Decreased ALB Groups in PLA Patients.**

| Indicators | PLA patients with normal ALB  (n=11) | PLA patients with decreased ALB  (n=86) | *p*-value |
| --- | --- | --- | --- |
| Gender, male | 5.0 (45.4%) | 55.0 (57.8%) | 0.431 |
| Age, years | 67.0±18.4 | 61.0±13.5 | 0.175 |
| Iron (μg/dL) | 40.0 (20.0,64.0) | 21.0 (13.0,31.0) | **0.008** |
| TF (mg/dL) | 190.5 (182.8,203.3) | 131.0 (110.0,156.0) | **<0.001** |
| TIBC (μg/dL) | 247.5 (237.3,264.3) | 170.0 (143.0,203.0) | **<0.001** |
| Ferritin (ng/mL) | 523.0 (205.0,931.0) | 548.0 (282.5,981.5) | 0.578 |
| RBC (m/μL) | 3.9 (3.4,4.1) | 3.2 (2.8,3.5) | **0.004** |
| HGB (g/dL) | 11.0 (9.5,12.8) | 9.2 (7.7,10.2) | **0.008** |
| HCT (%) | 32.9 (29.0,36.7) | 28.2 (25.0,31.0) | **0.005** |
| WBC (K/μL) | 7.2 (6.8,9.4) | 12.3 (8.6,16.9) | **0.006** |
| NEUT% (%) | 67.9 (62.5,84.0) | 83.0 (74.7,89.0) | **0.031** |
| MONO% (%) | 8.3 (5.3,11.0) | 5.0 (3.0,7.1) | **0.018** |
| ALT (IU/L) | 36 (20,248) | 33 (19,64) | 0.450 |
| AST (IU/L) | 33.0 (16.0,63.0) | 32.0 (22.0,53.0) | 0.800 |
| ALP (IU/L) | 112.0 (80.0,187.0) | 180.0 (116.0,328.0) | **0.043** |
| TBIL (mg/dL) | 0.6 (0.3,1.6) | 0.8 (0.4,1.5) | 0.783 |
| LDH (IU/L) | 253.5 (213.8,283.3) | 224.5 (168.3,285.5) | 0.458 |
| ALB (g/dL) | 3.7±0.2 | 2.6±0.4 | **<0.001** |
| PLT (K/μL) | 279.0(182.0,322.0) | 282.0(126.0,440.0) | 0.860 |
| INR (-) | 1.3(1.1,1.5) | 1.3(1.2,1.5) | 0.277 |
| PT (sec) | 13.5(12.5,15.8) | 14.7(13.5,16.8) | 0.158 |
| APTT (sec) | 28.3(26.2,31.9) | 30.5(28.1,34.6) | 0.078 |
| BUN (mg/dL) | 14.0(10.0,24.0) | 15.0(10.0,24.0) | 0.930 |
| CR (mg/dL) | 0.9(0.7,0.9) | 0.9(0.7,1.3) | 0.996 |
| GLU (mg/dL) | 105.0(96.0,143.0) | 129.0(102.0,169.0) | 0.168 |

Note: TF, transferrin; TIBC, total iron bind capacity; RBC, red blood cell; HGB, hemoglobin; HCT, hematocrit; WBC, white blood cell; NEUT%, neutrophil ratio; MONO%, monocyte ratio; ALT, alanine aminotransferase; AST, aspartate transaminase; ALP, alkaline phosphatase; TBIL, total bilirubin; LDH, lactate dehydrogenase; ALB, albumin; PLT, platelet; INR, international normalized ratio; PT, prothrombin time; APTT, activated partial thromboplastin time; BUN, blood urea nitrogen; CR, creatinine; GLU, glucose.

**Table S14. Comparison of Clinical Characteristics Between Normal and Increased PT Groups in PLA Patients.**

| Indicators | PLA patients with normal PT  (n=14) | PLA patients with increased PT  (n=101) | *p*-value |
| --- | --- | --- | --- |
| Gender, male | 5.0 (35.7%) | 61.0 (60.3%) | 0.080 |
| Age, years | 70.0±11.4 | 61.0±13.7 | **0.023** |
| Iron (μg/dL) | 35.0 (15.5,55.5) | 20.0 (12.0,30.0) | **0.008** |
| TF (mg/dL) | 162.6±43.7 | 141.5±44.3 | 0.136 |
| TIBC (μg/dL) | 211.6±56.8 | 183.9±57.5 | 0.133 |
| Ferritin (ng/mL) | 420.5 (271.5,718.0) | 593.5 (279.5,1027.3) | 0.578 |
| RBC (m/μL) | 3.3 (3.0,3.9) | 3.2 (2.8,3.6) | **0.004** |
| HGB (g/dL) | 9.5 (8.8,11.1) | 9.2 (7.7,10.3) | **0.008** |
| HCT (%) | 29.7 (27.1,34.8) | 28.1 (24.8,31.4) | **0.005** |
| WBC (K/μL) | 10.2 (7.5,12.0) | 11.9 (7.5,16.6) | **0.006** |
| NEUT% (%) | 78.7 (64.3,90.4) | 83.0 (74.7,88.0) | **0.031** |
| MONO% (%) | 4.9 (3.5,7.1) | 5.2 (3.1,7.7) | **0.018** |
| ALT (IU/L) | 35.0 (12.5,98.5) | 34.0 (19.0,64.0) | 0.450 |
| AST (IU/L) | 34.0 (16.5,101.0) | 32.5 (22.0,53.0) | 0.800 |
| ALP (IU/L) | 141.0 (92.0,185.0) | 176.5 (116.3,309.0) | **0.043** |
| TBIL (mg/dL) | 0.9 (0.3,2.1) | 0.7 (0.4,1.4) | 0.783 |
| LDH (IU/L) | 227.5 (172.3,248.0) | 222.0 (171.3,269.0) | 0.458 |
| ALB (g/dL) | 3.0±0.6 | 2.7±1.4 | 0.051 |
| PLT (K/μL) | 152.3 (92.3,327.3) | 298.0 (174.0,426.5) | 0.860 |
| INR (-) | 1.1 (1.1,1.1) | 1.4 (1.2,1.6) | 0.277 |
| PT (sec) | 11.9 (11.4,12.2) | 14.8 (13.7,16.9) | 0.158 |
| APTT (sec) | 27.1 (25.1,31.5) | 30.8 (28.4,34.8) | 0.078 |
| BUN (mg/dL) | 23.5 (14.0,63.8) | 14.0 (10.0,21.0) | 0.930 |
| CR (mg/dL) | 1.1 (0.8,3.6) | 0.8 (0.6,1.1) | 0.996 |
| GLU (mg/dL) | 125.5 (86.3,196.8) | 120.0 (96.0,155.5) | 0.168 |

Note: TF, transferrin; TIBC, total iron bind capacity; RBC, red blood cell; HGB, hemoglobin; HCT, hematocrit; WBC, white blood cell; NEUT%, neutrophil ratio; MONO%, monocyte ratio; ALT, alanine aminotransferase; AST, aspartate transaminase; ALP, alkaline phosphatase; TBIL, total bilirubin; LDH, lactate dehydrogenase; ALB, albumin; PLT, platelet; INR, international normalized ratio; PT, prothrombin time; APTT, activated partial thromboplastin time; BUN, blood urea nitrogen; CR, creatinine; GLU, glucose.

**Table S15. Comparison of Clinical Characteristics Between Normal and Increased WBC Groups in PLA Patients.**

| Indicators | PLA patients with normal WBC  (n=61) | PLA patients with increased WBC  (n=64) | *p*-value |
| --- | --- | --- | --- |
| Gender, male | 35.0 (57.3%) | 39.0 (60.9%) | 0.686 |
| Age, years | 62.0±13.4 | 61.0±13.7 | 0.760 |
| Iron (μg/dL) | 26.0 (17.0,40.0) | 20.0 (11.0,27.5) | **0.007** |
| TF (mg/dL) | 160.9±49.1 | 132.5±34.1 | **0.001** |
| TIBC (μg/dL) | 209.1±63.8 | 172..2±44.3 | **0.001** |
| Ferritin (ng/mL) | 340.0 (179.0,828.0) | 761.0 (340.0,1077.0) | **0.002** |
| RBC (m/μL) | 3.2 (2.8,3.7) | 3.3 (3.0,3.6) | 0.888 |
| HGB (g/dL) | 9.2 (7.7,10.6) | 9.4 (8.4,10.3) | 0.558 |
| HCT (%) | 28.9±5.6 | 29.4±5.3 | 0.607 |
| WBC (K/μL) | 7.3 (5.2,9.4) | 15.7 (12.7,19.2) | **<0.001** |
| NEUT% (%) | 74.4±12.3 | 84.0±8.3 | **<0.001** |
| MONO% (%) | 5.9 (4.2,8.9) | 4.5 (2.8,6.9) | **0.010** |
| ALT (IU/L) | 34.0 (18.3,63.5) | 31.0 (17.0,66.5) | 0.925 |
| AST (IU/L) | 28.0 (19.0,49.5) | 34.0 (22.8,58.8) | 0.262 |
| ALP (IU/L) | 134.0 (90.5,215.8) | 207.0 (125.0,318.3) | **0.008** |
| TBIL (mg/dL) | 0.6 (0.3,1.2) | 1.0 (0.5,1.6) | **0.007** |
| LDH (IU/L) | 214.0 (153.0,248.0) | 247.0 (192.0,325.0) | **0.020** |
| ALB (g/dL) | 3.0±0.5 | 2.5±0.4 | **<0.001** |
| PLT (K/μL) | 225.0 (121.5,343.5) | 320.0(184.3,465.3) | **0.009** |
| INR (-) | 1.3 (1.2,1.5) | 1.3 (1.2,1.5) | 0.429 |
| PT (sec) | 14.6 (13.1,16.2) | 14.7 (13.4,16.5) | 0.317 |
| APTT (sec) | 30.6 (28.0,34.3) | 29.9 (27.8,32.7) | 0.634 |
| BUN (mg/dL) | 13.0 (9.0,20.3) | 17.0 (10.3,24.8) | 0.094 |
| CR (mg/dL) | 0.8 (0.6,1.1) | 0.9 (0.7,1.3) | 0.362 |
| GLU (mg/dL) | 118.5 (99.0,154.3) | 120.5 (93.8,184.3) | 0.427 |

Note: TF, transferrin; TIBC, total iron bind capacity; RBC, red blood cell; HGB, hemoglobin; HCT, hematocrit; WBC, white blood cell; NEUT%, neutrophil ratio; MONO%, monocyte ratio; ALT, alanine aminotransferase; AST, aspartate transaminase; ALP, alkaline phosphatase; TBIL, total bilirubin; LDH, lactate dehydrogenase; ALB, albumin; PLT, platelet; INR, international normalized ratio; PT, prothrombin time; APTT, activated partial thromboplastin time; BUN, blood urea nitrogen; CR, creatinine; GLU, glucose.

**Table S16. Comparison of Clinical Characteristics Between Normal and Increased NEUT% Groups in PLA Patients.**

| Indicators | PLA patients with normal NEUT%  (n=21) | PLA patients with increased NEUT%  (n=86) | *p*-value |
| --- | --- | --- | --- |
| Gender, male | 14.0 (66.6%) | 48.0 (55.8%) | 0.366 |
| Age, years | 65.0±13.9 | 61.0±14.1 | 0.239 |
| Iron (μg/dL) | 31.0 (20.0,53.0) | 19.5 (12.0,29.0) | **0.007** |
| TF (mg/dL) | 171.4±38.9 | 143.2±45.0 | **0.016** |
| TIBC (μg/dL) | 222.9±50.5 | 186.2±58.5 | **0.016** |
| Ferritin (ng/mL) | 400.5 (190.8,888.5) | 617.0 (292.5,958.0) | 0.165 |
| RBC (m/μL) | 3.2 (2.9,3.7) | 3.3 (3.0,3.6) | 0.805 |
| HGB (g/dL) | 9.1 (7.9,11.0) | 9.3 (7.9,10.4) | 0.922 |
| HCT (%) | 27.7 (25.7,33.4) | 28.9 (25.6,31.6) | 0.934 |
| WBC (K/μL) | 7.6±2.9 | 13.2±6.7 | **<0.001** |
| NEUT% (%) | 63.8 (57.4,66.9) | 84.0 (78.3,89.0) | **<0.001** |
| MONO% (%) | 8.6±3.8 | 5.0±2.9 | **<0.001** |
| ALT (IU/L) | 24.0 (16.0,50.0) | 36.0 (19.0,70.0) | 0.110 |
| AST (IU/L) | 22.0 (18.0,46.0) | 37.0 (23.0,70.0) | **0.016** |
| ALP (IU/L) | 136.0 (89.0,328.0) | 161.0 (112.0,272.0) | 0.429 |
| TBIL (mg/dL) | 0.6 (0.3,1.2) | 0.7 (0.4,1.3) | 0.224 |
| LDH (IU/L) | 237.5 (150.3,254.8) | 223.0 (176.0,270.0) | 0.654 |
| ALB (g/dL) | 3.0±0.6 | 2.7±0.5 | **0.027** |
| PLT (K/μL) | 263.0 (99.0,372.5) | 281.5 (138.0,416.8) | 0.551 |
| INR (-) | 1.3 (1.1,1.5) | 1.4 (1.2,1.5) | 0.514 |
| PT (sec) | 13.9 (12.5,16.3) | 15.0 (13.4,16.8) | 0.229 |
| APTT (sec) | 31.1 (27.7,36.0) | 30.0 (27.8,33.9) | 0.989 |
| BUN (mg/dL) | 13.0 (12.0,31.5) | 15.0 (10.0,21.8) | 0.606 |
| CR (mg/dL) | 0.9 (0.8,1.5) | 0.8 (0.6,1.2) | 0.304 |
| GLU (mg/dL) | 112.5 (97.5,142.0) | 119.0 (93.0,167.5) | 0.598 |

Note: TF, transferrin; TIBC, total iron bind capacity; RBC, red blood cell; HGB, hemoglobin; HCT, hematocrit; WBC, white blood cell; NEUT%, neutrophil ratio; MONO%, monocyte ratio; ALT, alanine aminotransferase; AST, aspartate transaminase; ALP, alkaline phosphatase; TBIL, total bilirubin; LDH, lactate dehydrogenase; ALB, albumin; PLT, platelet; INR, international normalized ratio; PT, prothrombin time; APTT, activated partial thromboplastin time; BUN, blood urea nitrogen; CR, creatinine; GLU, glucose.

**Table S17. Comparison of Clinical Characteristics Between Normal and Increased MONO% Groups in PLA Patients.**

| Indicators | PLA patients with normal MONO%  (n=84) | PLA patients with increased MONO%  (n=9) | *p*-value |
| --- | --- | --- | --- |
| Gender, male | 57.0 (58.1%) | 5.0 (55.5%) | 0.879 |
| Age, years | 61.0±14.2 | 64.0±13.4 | 0.603 |
| Iron (μg/dL) | 21.0 (12.8,33.0) | 20.0 (11.5,31.5) | 0.735 |
| TF (mg/dL) | 145.0 (117.0,172.3) | 185.0 (117.0,192.5) | 0.357 |
| TIBC (μg/dL) | 189.0 (152.0,224.0) | 241.0 (152.0,250.0) | 0.357 |
| Ferritin (ng/mL) | 554.0 (281.8,924.0) | 384.0 (217.0,1770.5) | 0.952 |
| RBC (m/μL) | 3.3 (3.0,3.6) | 3.5 (2.8,4.5) | 0.479 |
| HGB (g/dL) | 9.2 (7.9,10.4) | 9.5 (8.2,11.9) | 0.409 |
| HCT (%) | 28.7 (25.7,31.6) | 29.0 (25.1,36.9) | 0.598 |
| WBC (K/μL) | 11.1 (7.2,16.3) | 7.4 (5.2,16.1) | 0.203 |
| NEUT% (%) | 83.0 (74.9,88.1) | 66.7 (53.8,73.1) | **<0.001** |
| MONO% (%) | 5.0±2.7 | 13.0±1.4 | **<0.001** |
| ALT (IU/L) | 35.0 (19.0,66.5) | 43.0 (16.5,61.5) | 0.961 |
| AST (IU/L) | 34.0 (22.0,59.5) | 37.0 (18.0,48.0) | 0.605 |
| ALP (IU/L) | 160.0 (105.5,279.0) | 134.0 (101.5,190.5) | 0.410 |
| TBIL (mg/dL) | 0.7 (0.4,1.3) | 1.0 (0.4,1.8) | 0.826 |
| LDH (IU/L) | 227.5 (180.3,266.5) | 223.0 (120.5,303.5) | 0.359 |
| ALB (g/dL) | 2.8±0.6 | 3.0±0.4 | 0.311 |
| PLT (K/μL) | 281.5 (127.5,416.8) | 220.0 (133.0,356.0) | 0.515 |
| INR (-) | 1.3 (1.2,1.5) | 1.4 (1.3,1.7) | 0.151 |
| PT (sec) | 14.7 (13.2,16.6) | 16.0 (13.8,18.5) | 0.292 |
| APTT (sec) | 30.1 (27.8,33.8) | 31.0 (28.4,36.2) | 0.628 |
| BUN (mg/dL) | 15.0 (10.0,22.0) | 12.0 (11.5,28.0) | 0.858 |
| CR (mg/dL) | 0.8 (0.6,1.2) | 1.0 (0.9,1.1) | 0.362 |
| GLU (mg/dL) | 118.0 (93.0,163.0) | 121.0 (95.0,137.0) | 0.652 |

Note: TF, transferrin; TIBC, total iron bind capacity; RBC, red blood cell; HGB, hemoglobin; HCT, hematocrit; WBC, white blood cell; NEUT%, neutrophil ratio; MONO%, monocyte ratio; ALT, alanine aminotransferase; AST, aspartate transaminase; ALP, alkaline phosphatase; TBIL, total bilirubin; LDH, lactate dehydrogenase; ALB, albumin; PLT, platelet; INR, international normalized ratio; PT, prothrombin time; APTT, activated partial thromboplastin time; BUN, blood urea nitrogen; CR, creatinine; GLU, glucose.

**Table S18. Pairwise Comparisons of Indicators Among Three PLA Subgroups.**

| **Indicators** | **Mild vs. Moderate  *p*-value** | **Mild vs. Severe  *p*-value** | **Moderate vs. Severe  *p*-value** |
| --- | --- | --- | --- |
| Iron (μg/dL) | 0.097 | **0.008** | 0.485 |
| Ferritin (ng/mL) | **0.044** | **0.002** | 0.362 |
| WBC (K/μL) | **<0.001** | **<0.001** | **0.001** |
| NEUT% (%) | **0.045** | **<0.001** | 0.062 |
| ALT (IU/L) | **0.013** | **<0.001** | **<0.001** |
| AST (IU/L) | **0.013** | **<0.001** | **0.001** |
| ALP (IU/L) | **<0.001** | **0.002** | >0.999 |
| TBIL (mg/dL) | **0.045** | **0.001** | 0.148 |
| ALB (g/dL) | **0.011** | 0.105 | 0.516 |
| ALBI score | **0.002** | **0.005** | 0.814 |
| APRI score | 0.162 | **0.001** | 0.067 |

Note: WBC, white blood cell; NEUT%, neutrophil ratio; ALT, alanine aminotransferase; AST, aspartate transaminase; ALP, alkaline phosphatase; TBIL, total bilirubin; ALB, albumin; ALBI, Albumin - Bilirubin Score; APRI, Aspartate Aminotransferase to Platelet Ratio Index.

**Table S19. Correlation between iron metabolism indicators and liver function or systemic inflammation levels in patients with BLA.**

| Indicators | Iron | | TF | | TIBC | | Ferritin | | RBC | | HGB | | HCT | |
| --- | --- | --- | --- | --- | --- | --- | --- | --- | --- | --- | --- | --- | --- | --- |
|  | r | *p* | r | *p* | r | *p* | r | *p* | r | *p* | r | *p* | r | *p* |
| WBC | -0.089 | 0.494 | -0.13 | 0.330 | -0.130 | 0.330 | 0.379 | **0.003** | 0.020 | 0.879 | 0.212 | 0.099 | 0.187 | 0.146 |
| NEUT% | -0.094 | 0.512 | -0.449 | **0.001** | -0.449 | **0.001** | 0.349 | **0.014** | -0.222 | 0.113 | -0.009 | 0.947 | -0.053 | 0.710 |
| MONO% | -0.107 | 0.453 | 0.339 | **0.018** | 0.339 | **0.018** | -0.239 | 0.098 | 0.241 | 0.086 | 0.080 | 0.575 | 0.069 | 0.626 |
| ALT | -0.170 | 0.198 | 0.279 | **0.037** | 0.279 | **0.037** | 0.183 | 0.172 | 0.280 | **0.030** | 0.389 | **0.002** | 0.372 | **0.003** |
| AST | -0.098 | 0.461 | 0.134 | 0.326 | 0.134 | 0.326 | 0.210 | 0.116 | 0.175 | 0.181 | 0.315 | **0.014** | 0.311 | **0.015** |
| ALP | 0.142 | 0.285 | -0.301 | **0.024** | -0.301 | **0.024** | 0.117 | 0.388 | -0.212 | 0.104 | -0.196 | 0.134 | -0.231 | 0.076 |
| LDH | 0.090 | 0.523 | -0.022 | 0.880 | -0.022 | 0.880 | 0.465 | **0.001** | -0.105 | 0.454 | 0.086 | 0.540 | 0.108 | 0.441 |
| ALB | -0.240 | 0.078 | 0.538 | **<0.001** | 0.538 | **<0.001** | -0.121 | 0.388 | 0.340 | **0.010** | 0.272 | **0.042** | 0.313 | **0.019** |

Note: BLA, bacterial liver abscess; WBC, white blood cell; NEUT%, neutrophil ratio; MONO%, monocyte ratio; ALT, alanine aminotransferase; AST, aspartate transaminase; ALP, alkaline phosphatase; LDH, lactate dehydrogenase; ALB, albumin; TF, transferrin; TIBC, total iron bind capacity; RBC, red blood cell; HGB, hemoglobin; HCT, hematocrit; r, related coefficient; *p*, *p*-value.
