# Supplementary figures and images for: Iron Metabolism Disorders Associated With the Severity of Pyogenic Liver Abscess
Source: Food Sci Nutr. 2025 Nov 21;13(11):e71195. doi: 10.1002/fsn3.71195 (PMC12635943; doi:10.1002/fsn3.71195)

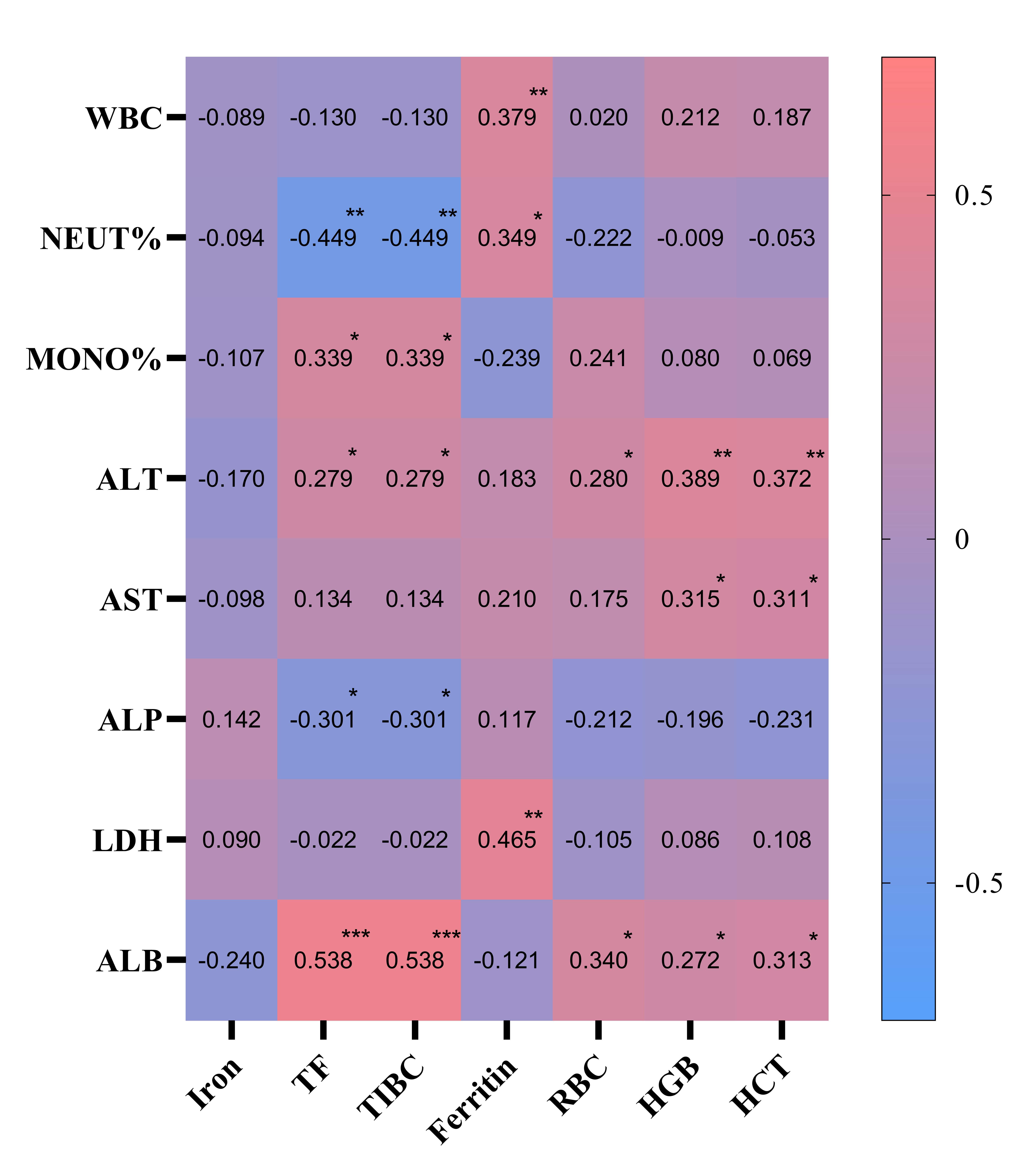

Supplement: Supplementary file 1 — Figure S1: The distribution of pathogens in patients with PLA. Figure S2: Correlation heat map of iron metabolism indicators with liver function and systemic inflammation level in patients with BLA. Figure S3: Receiver operating characteristic (ROC) curve analysis of ferritin for detecting bacterial liver abscess (BLA). Table S1: Reference intervals for clinical indicators. Table S2: Difference analysis of indicators among three PLA subgroups before and after imputation using the mean value. Table S3: Difference analysis of the ferritin subgroups before and after imputation using the mean value. Table S4: Correlation between iron metabolism indicators and liver function or systemic inflammation levels in patients with PLA. Table S5: Comparison of clinical characteristics between MELD score ≤ 9 and MELD score > 9 groups in PLA patients. Table S6: Comparison of clinical characteristics between ALBI score ≤ −1.39 and ALBI score > −1.39 groups in PLA patients. Table S7: Comparison of clinical characteristics between APRI score ≤ 2 and APRI score > 2 groups in PLA patients. Table S8: Comparison of clinical characteristics between normal and increased ALT groups in PLA patients. Table S9: Comparison of clinical characteristics between normal and increased AST groups in PLA patients. Table S10: Comparison of clinical characteristics between normal and increased ALP groups in PLA patients. Table S11: Comparison of clinical characteristics between normal and increased LDH groups in PLA patients. Table S12: Comparison of clinical characteristics between normal and increased TBIL groups in PLA patients. Table S13: Comparison of clinical characteristics between normal and decreased ALB groups in PLA patients. Table S14: Comparison of clinical characteristics between normal and increased PT groups in PLA patients. Table S15: Comparison of clinical characteristics between normal and increased WBC groups in PLA patients. Table S16: Comparison of clinical characteristics between no [file FSN3-13-e71195-s001.zip › Figure_S2.tif]

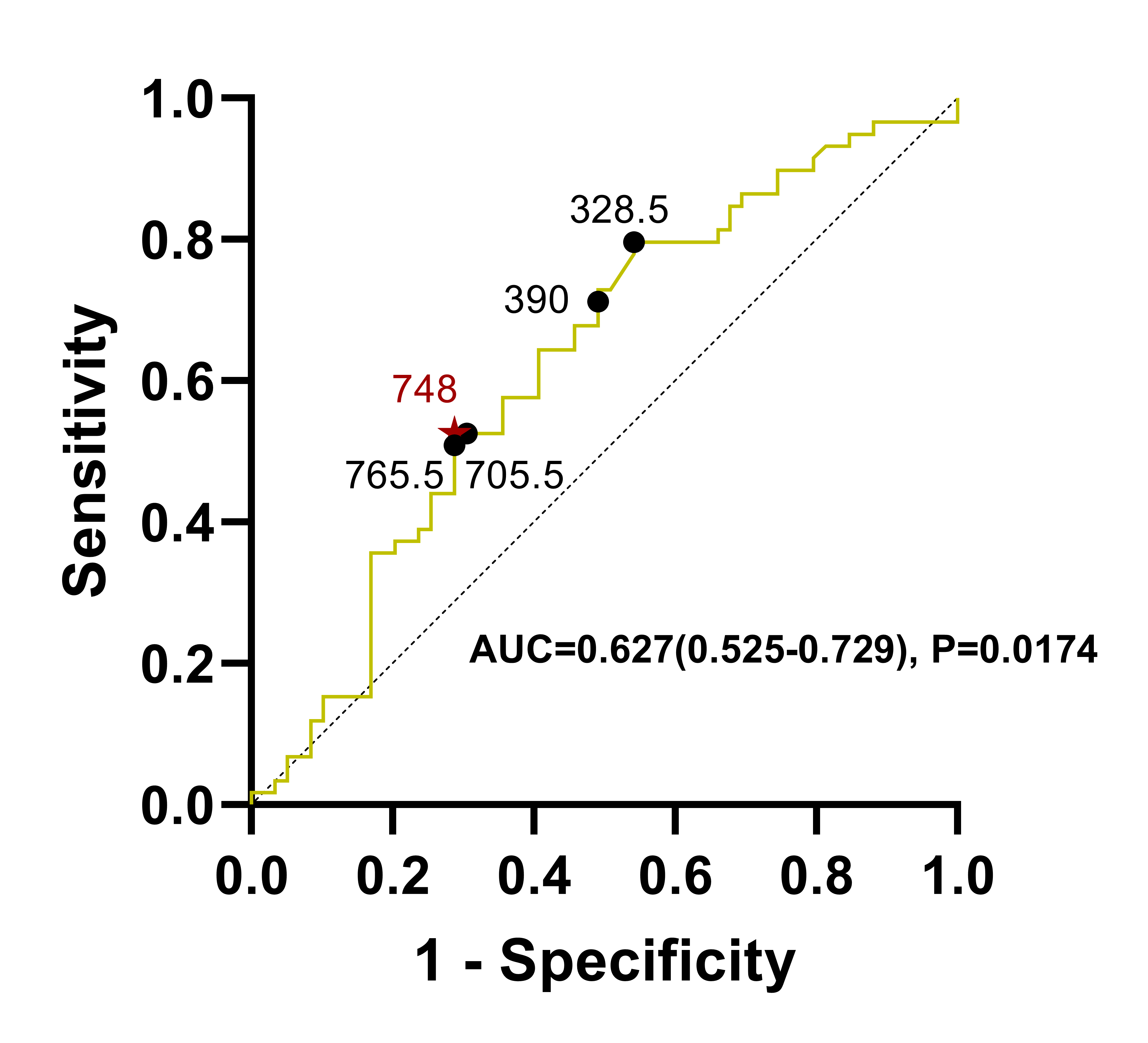

Supplement: Supplementary file 1 — Figure S1: The distribution of pathogens in patients with PLA. Figure S2: Correlation heat map of iron metabolism indicators with liver function and systemic inflammation level in patients with BLA. Figure S3: Receiver operating characteristic (ROC) curve analysis of ferritin for detecting bacterial liver abscess (BLA). Table S1: Reference intervals for clinical indicators. Table S2: Difference analysis of indicators among three PLA subgroups before and after imputation using the mean value. Table S3: Difference analysis of the ferritin subgroups before and after imputation using the mean value. Table S4: Correlation between iron metabolism indicators and liver function or systemic inflammation levels in patients with PLA. Table S5: Comparison of clinical characteristics between MELD score ≤ 9 and MELD score > 9 groups in PLA patients. Table S6: Comparison of clinical characteristics between ALBI score ≤ −1.39 and ALBI score > −1.39 groups in PLA patients. Table S7: Comparison of clinical characteristics between APRI score ≤ 2 and APRI score > 2 groups in PLA patients. Table S8: Comparison of clinical characteristics between normal and increased ALT groups in PLA patients. Table S9: Comparison of clinical characteristics between normal and increased AST groups in PLA patients. Table S10: Comparison of clinical characteristics between normal and increased ALP groups in PLA patients. Table S11: Comparison of clinical characteristics between normal and increased LDH groups in PLA patients. Table S12: Comparison of clinical characteristics between normal and increased TBIL groups in PLA patients. Table S13: Comparison of clinical characteristics between normal and decreased ALB groups in PLA patients. Table S14: Comparison of clinical characteristics between normal and increased PT groups in PLA patients. Table S15: Comparison of clinical characteristics between normal and increased WBC groups in PLA patients. Table S16: Comparison of clinical characteristics between no [file FSN3-13-e71195-s001.zip › Figure_S3.tif]
